# Nanoparticle‐Delivered siRNA Targeting NSUN4 Relieves Systemic Lupus Erythematosus through Declining Mitophagy‐Mediated CD8+T Cell Exhaustion

**DOI:** 10.1002/mco2.70311

**Published:** 2025-08-03

**Authors:** Bincheng Ren, Kaini He, Ning Wei, Shanshan Liu, Xiaoguang Cui, Xin Yang, Xiaojing Cheng, Tian Tian, Ru Gu, Xueyi Li

**Affiliations:** ^1^ Department of Rheumatology and Immunology The Second Affiliated Hospital of Xi'an Jiaotong University Xi'an China; ^2^ Department of Gastroenterology The Second Affiliated Hospital of Xi'an Jiaotong University Xi'an China; ^3^ College of Animal Science and Technology Northwest A&F University Yangling China; ^4^ Department of Anesthesiology The Second Affiliated Hospital of Xi'an Jiaotong University Xi'an China

**Keywords:** CD8+T cell mitophagy and exhaustion, nanoparticle‐delivered siRNA, NSUN4, single‐cell RNA sequencing and m5C sequencing, systemic lupus erythematosus

## Abstract

5‐Methylcytosine modification (m5C) is an important posttranscriptional regulatory mechanism of gene expression. Exhausted CD8+T cells contribute to the development of many major diseases; however, their exact role and relationship to m5C in systemic lupus erythematosus (SLE) remain unknown. In this study, we identified a CD7^high^CD74^high^ CD8+T subgroup that were robustly expanded in SLE patients through single‐cell transcriptome sequencing (scRNA‐seq). CD7^high^CD74^high^ CD8+T cells displayed exhausted features and exhibited a superior diagnostic value in SLE. Then, we explored the m5C landscape of SLE patients by performing m5C epitranscriptome sequencing (m5C‐seq). ScRNA‐seq and m5C‐seq were conjointly analyzed to screen m5C‐related therapeutic targets for SLE, and NOP2/Sun RNA methyltransferase 4 (NSUN4) was identified as a key regulator of SLE pathogenesis. Knockdown of NSUN4 downregulated CD74 expression via reduction of m5C and suppressed CD8+T cell exhaustion by declining CD44/mTOR (mechanistic target of rapamycin kinase)‐mediated mitophagy. Finally, we verified that nanoparticle‐delivered siRNA against *Nusn4* decreased autoimmune reaction kidney damage in both spontaneous and pristane‐induced SLE mouse models. In conclusion, we identify an exhausted CD7^high^CD74^high^ CD8+T cell subset and propose the crucial role of NSUN4/CD74‐induced dysregulation of mitophagy in SLE pathogenesis, and targeting NSUN4 is a promising treatment strategy for SLE patients.

## Graphical abstract

CD74^high^CD7^high^ exhausted CD8+T cells were characterized by ScRNA‐seq, and their association with SLE diagnostic indicators was evaluated. NSUN4 was screened as a therapeutic target by integrative analysis of scRNA‐seq and m5C‐seq, and its role in mitochondrial dysfunction was investigated in cultured CD8+T cells in vitro. The therapeutic effect of LPH‐nanoparticle delivered *Nsun4*–siRNA was confirmed in spontaneous lupus mice and pristane‐induced SLE mice in vivo.

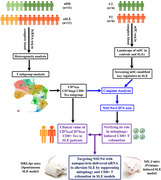

## 1 Introduction

Systemic lupus erythematosus (SLE) is a chronic autoimmune connective tissue disease that is more common in young women than in young men. SLE has the characteristics of hidden onset, long course, and poor prognosis similar to other autoimmune diseases, but it is more extreme. Moreover, SLE affects multiple organs and tissues, including the derma, muscle, skeleton, and kidney, as well as organs and glands of the digestive, reproductive, circulatory, nervous, and endocrine systems. To date, the etiology of SLE is not fully understood, and it cannot be cured but controlled through lifelong medication [1, 2]. Under perturbations in self‐tolerance, B lymphocytes are excessively activated and produce a large number of autoantibodies to combine with the corresponding autoantigens to form corresponding immune complexes, leading to acute and chronic inflammation, cell damage, and tissue necrosis [3]. Whereas, it is gradually admitted that T‐cell abnormalities are also important in induction of autoimmunity and responsible for organ damage, among which effector functions of phenotypic changed CD8+T cells (also known as cytotoxic T cells) play a central role. Evidence has revealed that exhausted subpopulations of CD8+T cells are increased in patients and may serve as a tolerance mechanism in SLE, providing a potential therapeutic tool for patients [4, 5, 6]. Therefore, identifying which CD8+ T subpopulations are required for SLE progression and revealing how these subpopulations are generated are critical for elucidating the etiology of SLE and developing reliable therapeutic strategies.

ScRNA‐seq has been increasingly applied to the exploration of landscapes of the immune microenvironment in major human diseases including SLE. During the past several years, several scRNA‐seq studies have revealed the heterogeneity and complexity of the cell components in the microenvironment of SLE patients, identified a few associated immune cell subsets and molecular biomarkers, and attempted preclinical therapy research based on immune molecular targets. For example, a large‐scale multiplexed scRNA sequencing study of 1.2 million adult human peripheral blood mononuclear cells (PBMCs) indicated that SLE monocytes exhibited high expression of interferon (IFN)‐stimulated genes (ISGs), the number of naïve CD4+T cells was reduced and GZMH+CD8+ T cells were expanded in SLE patients [7]. Children with SLE also display high heterogeneity and altered composition of PBMCs, such as increased exhausted CD4+T cells and ISGhi_Granzyme K+_acCD8+ T cells [8]. ScRNA‐seq data of cutaneous lesions revealed that expanded chemokine ligand 20+ keratinocytes, C‐X‐C chemokine ligand 1+ fibroblasts, ISG^high^ CD4/CD8 T cells, and ISG^high^ plasma cell/pDC/natural killer (NK) subclusters were identified in discoid LE (DLE) and SLE patients, compared with healthy controls (HCs), and higher cell communication scores between some cell types were also observed in DLE and SLE patients [9]. A recent scRNA‐seq assay combined with sequencing assay for transposase‐accessible chromatin (ATAC‐seq) performed in peripheral CD4+ T cells from SLE patients and HCs identified a CCR7^low^CD74^high^ Treg subgroup in SLE patients, which revealed a novel contribution of exhausted T cells in SLE pathogenesis and showed the prospect of joint sequencing analyses in exploring the pathological mechanisms of SLE [10]. New associated PBMC subpopulations, especially B and T lymphocyte subgroups, are still being discovered to help us better understand the pathogenesis of SLE, so as to accelerate our pace of conquering SLE.

RNA methylation, mainly involving N6‐methyladenosine (m6A), 5‐methylcytosine (m5C), N1‐methyladenosine, and N7‐methylguanosine, represents an important class of RNA modification and is a highly relevant field of posttranscriptional regulation. Researchers have gradually uncovered the RNA methylation landscapes of many diseases through RNA methylation sequencing analyses (also named m5C antibody‐based methylated RIP [meRIP]‐seq or epitranscriptome sequencing) [11, 12]. The RNA methylation level is regulated by specific writers (methyltransferases) or erasers (demethylases), and then the fate of the methylated RNA, such as transportation, splicing, stabilization, and degradation, is handled by the readers (specific RNA binding proteins that recognize and bind to methylated RNA sequences) [13]. m6A modification profiling showed that the general level of m6A methylation was altered in SLE patients and m6A‐related RNAs might be involved in the pathogenesis of SLE [14]. Moreover, a few case reports or cellular explorations have demonstrated that m6A regulators, such as writers methyltransferase like 3/14, eraser Alkylation repair homolog protein 5, and readers YTH domain family member 2 and insulin like growth factor binding protein 3, are dysregulated in SLE PMBCs and regulate functions of CD4+T cell activation and effector T cell differentiation, providing promising therapeutic targets for SLE [15, 16, 17, 18, 19, 20]. m5C is the most common type of RNA methylation, second only to m6A. To date, it is largely unknown how m5C alterations in the SLE microenvironment and what are their association with SLE.

In this study, we explored the heterogeneities of blood cells and identified new subgroups of T cells associated with SLE pathogenesis by performing scRNA‐seq on 13 adult female SLE patients (group aSLE) and 11 healthy donors (group aHD) to explore the heterogeneity of SLE blood cells. 27 cell clusters (0–26) and 10 cell types were found in a total of 149,513 single cells from the 24 subjects. In‐depth T cell subgroup analysis screened an exhausted CD7^high^CD74^high^ CD8+T cell subgroup in No. 16 cluster that displayed exhausted features and exhibited a superior diagnostic value in SLE patients. Then, m5C epitranscriptome sequencing was performed in CBCs from another six SLE patients (group P_2_) and six HCs (group C_2_) to explore the m5C landscapes of control and SLE patients. Moreover, we performed a conjoint analysis of the scRNA transcriptome and m5C epitranscriptome to screen key regulators in the newly identified immune cell subgroups. SLE patients displayed a distinct landscape of m5C modification compared with controls, and the m5C writer NSUN4 was upregulated in SLE patients and played an important role in CD74 expression and CD74‐mediated mitophagy to contribute to the exhaustion of CD8+T cells.

## 2 Results

### 2.1 Total T Cells are Sharply Reduced but the Cluster 16 T Cell Subpopulation are Expanded in SLE Patients

**FIGURE 1:**
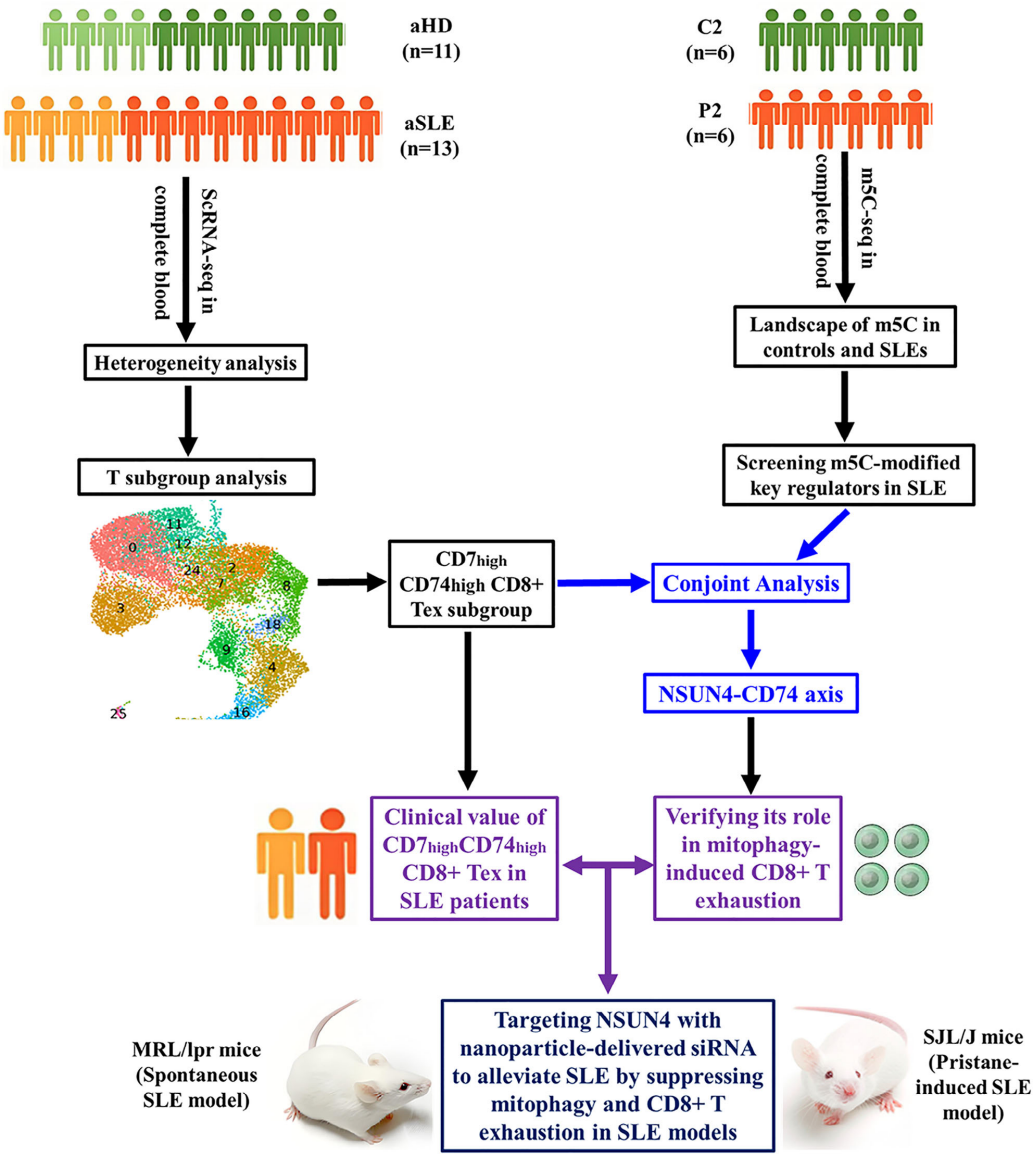
Over design of this study. C, healthy control; DEG, differentially expressed gene; HD, healthy donor; P, SLE patients; SLE, systemic lupus erythematosus.

**FIGURE 2:**
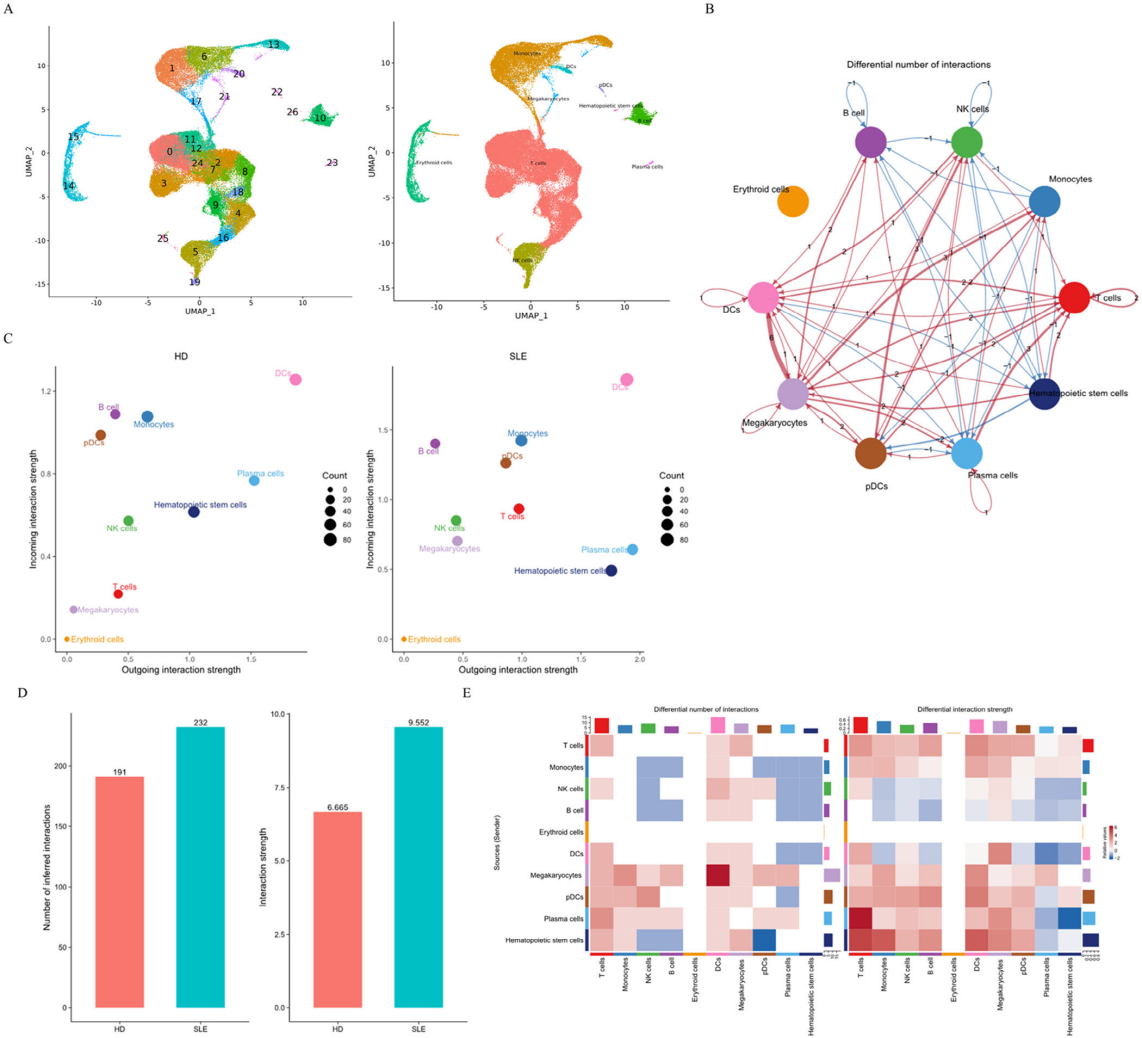
Deep UMAP assay in all clusters and cell–cell communications among all cell types. (A) UMAP assay for cell clusters (left panel) and cell types (right panel) of blood cells in overall samples. (B) Overall interaction numbers between every two cell types in all subjects. (C) Interaction strengths of every cell type between them as an incoming signaling and an outcoming signaling. (D) Total interfered interaction numbers of all cell types between HDs and SLEs. (F) Cross matrices about interaction numbers and interaction strengths between every two cell types respectively in HDs and SLEs.

To explore the heterogeneity of blood cells in SLE patients and their associations with SLE pathogenesis, we first performed single‐cell transcriptome sequencing of complete blood cells (CBCs) from 13 adult SLE patients (aSLE, four male and nine female) and 11 adult HCs (aHD, four male and seven female), followed by conjoint analysis with the m5C epitranscriptome in six SLE patients and six controls (Figure 1). The 24 CBC samples contained 157,741 cells. The Seurat R package (v 4.2.0) was applied for low‐quality cell filtration, standardization, and data merging, with the criterion of nUMIs (number of unique molecular identifiers) > 400, 0 < nGenes < 2500, mitoRatio < 0.2. Finally, we generated 149,513 single‐cell transcriptomes of healthy and SLE CBCs (Table S1). After principal component analysis (PCA), we used uniform manifold approximation and projection (UMAP) and t‐distributed stochastic neighbor embedding (t‐SNE) to perform UMAP nonlinear clustering and dimensionality reduction analyses respectively in overall samples and different groups. Seurat software was used to perform comparative difference analysis, including clustering, marker gene analysis, conservative marker gene analysis, and differential gene analysis under different conditions. According to unsupervised cluster analysis based on the proximity relationship between the similarity of single cell expression profiles, all cells were divided into 27 clusters (0–26) (Figures 2A and S1A and Table S1), among which clusters 0, 2, 3, 4, 7, 8, 9, 11, 12, 16, 18, 24, and 25 belonged to T cells; clusters 1, 6, 13, 17, and 21 belonged to monocytes; clusters 5 and 19 belonged to NK cells; cluster 10 belonged to B cells; clusters 14 and 15 belonged to erythroid cells; cluster 20 belonged to dendritic cells (DCs), cluster 22 belonged to plasmacytoid DCs (pDCs), cluster 23 belonged to plasma cells; and cluster 26 belonged to hematopoietic stem cells (Figures 2A and S1B). UMAP and t‐SNE analyses in different cell clusters at various cell cycle stages showed that, generally, all cell clusters were most abundant in the G1 stage, followed by the S stage, and least abundant in the G2/M stage (Figure S1C,D).

In SLE pathogenesis, B cells are responsible for generating autoimmune antibodies, whereas T cells play an important immunomodulatory role. Here, cell communications between the blood cells in aHD and SLE were analyzed and compared using the R package Cellchat, and the cell communication status under the two conditions was compared. In addition, using the R package Monocle 3 based on UMAP dimensionality reduction, we performed quasi temporal (also known as pseudotime) analyses from the root nodes of each cell group. The results showed that T cells, the population with the greatest reduction in SLE blood, displayed a moderate overall interaction score in all subjects (Figure 2B). In aHD, T cells displayed relatively low interaction numbers and strength, but significantly higher interaction numbers and strength in aSLEs (Figure 2C–E). Quasi temporal (pseudotime) analysis was performed using R package Monocle3 based on UMAP dimensionality reduction in overall blood cells or each cell type to further understand the derivative relationships among the clusters and cell types (Figure S3).

Then, the number and classification of the 27 clusters were compared in aHDs and aSLEs. Clusters 10 (total B cells) and 22 (total pDCs) were reduced in SLE patients, while clusters 1, 6 13, 17, and 21 (constituted to total monocytes) were all expanded; moreover, most subgroups of T cells, including clusters 0, 2, 3, 4, 7, 8, 9, 11, 12, 18, and 24, were decreased. It is worth mentioning that total T cells were obviously reduced, but cluster 16 was the only T cell subgroup that increased in SLE patients (43.5 vs. 199.8; Table S1), except for cluster 25 (10.1 vs. 18.2; Table S1).

### 2.2 Exhausted CD7^high^CD74^high^ T Cells in Cluster 16 are Associated with SLE Pathogenesis

**FIGURE 3:**
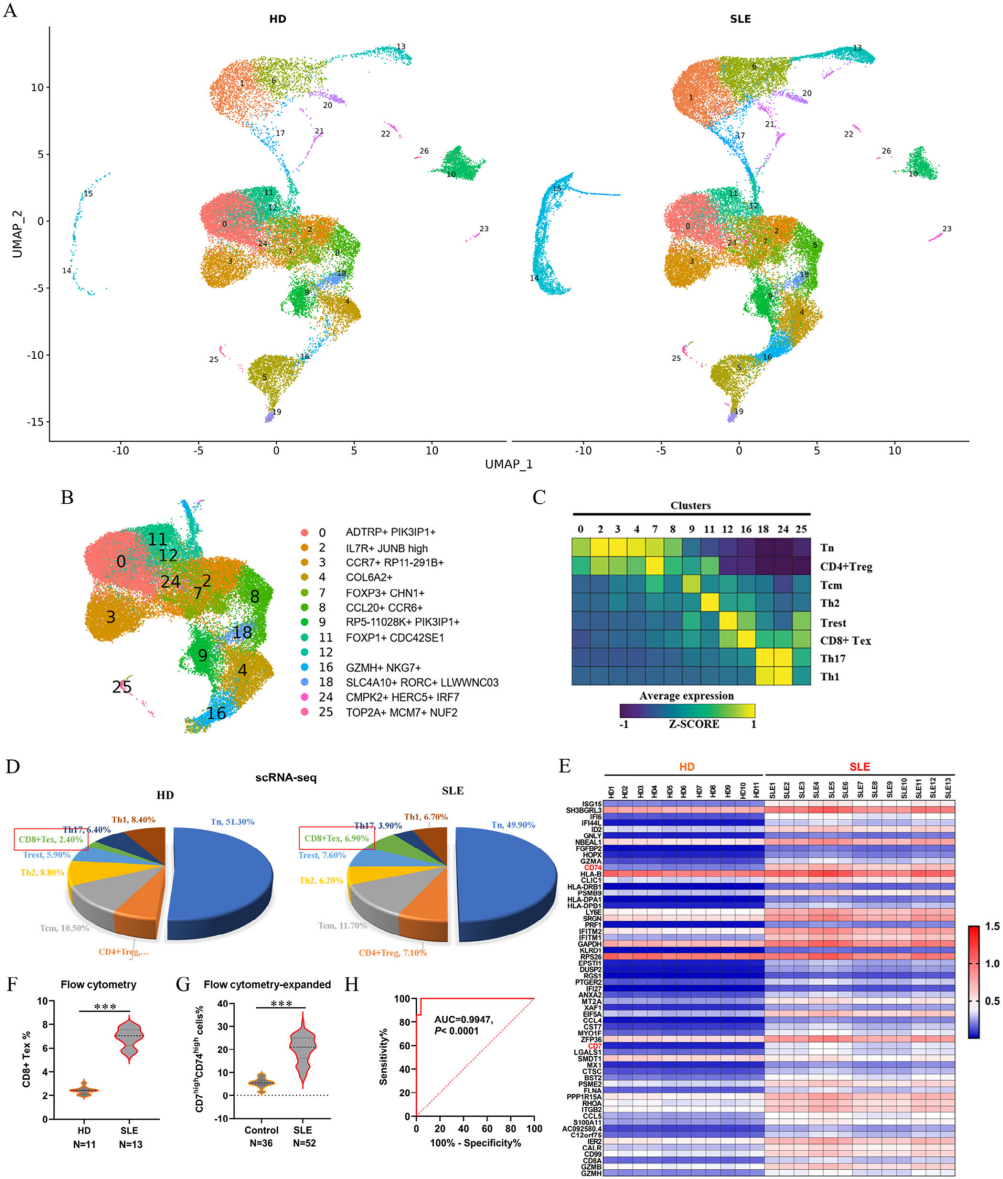
An exhausted CD7^high^CD74^high^ CD8+T cell subgroup was discovered in cluster 16 of SLE blood cells. (A) UMAP assay for cell clusters of blood cells in HDs and SLEs. (B) Annotation for the characteristic surface biomarkers of cell clusters in total T cells based on UMAP assay. (C) Annotation for the subgroups of total T cells based on the surface biomarkers. (D) The percentages of each T cell subgroup in HDs and SLEs, respectively. (E) Heatmap of DEGs in cluster 16 between HDs and SLEs. (F) Flow cytometry assay for the (F) percentages of CD8+ Tex cells/total T cells in the subjects enrolled in the scRNA‐seq assay and (G) CD7^high^CD74^high^ T cells/CD8+NKG7+ T cells in expanded population of controls (*N* = 36) and SLEs (*N* = 52), respectively. (H) Receiver operating characteristic curve (ROC) was used to evaluate the diagnostic sensitivity and specificity in 52 SLE patients. ***p < 0.01.

The role of CD4+T cells in SLE has been relatively well characterized: they produce excessive interleukin (IL)‐17, infiltrate tissues, and help B cells generate autoimmune antibodies to trigger autoimmune injury of tissues. However, the role of CD8+T cells in SLE remains unclear. Next, we performed deep analysis on T cell subgroups including clusters 0, 2, 3, 4, 7, 8, 9, 11, 12, 16, 18, 24, and 25. Subtype analysis indicated that they could be respectively classified to Tn (mainly composed of clusters 0, 2, 3, 4, and 8), CD4+ Treg (regulatory T cells, mainly composed of cluster 7, FOXP3+CHN1+, Forkhead box protein P3, and Chimerin 1 double positive), Tcm (Central memory T cells, mainly composed of cluster 9, PR5‐11028K+PIK3IP1+, pathogenesis‐related protein 5–11028K, and phosphoinositide‐3‐kinase‐interacting protein 1 double positive), Th2 (T helper cell type 2, mainly composed of cluster 11, FOXP1+CDC42SE1+, Forkhead box protein P1, and cell division cycle 42 small effector 1 double positive), Trest (resting T cells, mainly composed of clusters 12 and 25), CD8+ Tex (exhausted CD8+ T cells, mainly composed of cluster 16, GZMH+NKG7+, granzyme H, and natural killer cell granular protein 7 double positive), Th17 (T helper cell type 17 mainly composed of cluster 18 and a part of cluster 24), and Th1 (T helper cell type 1, mainly composed of a small portion of cluster 18 and other part of cluster 24) (Figure 3B,C). Cell counting assay based on scRNA‐seq showed that there were only about 2.4% of CD8+ Tex in T cells from HDs, but nearly 7% of CD8+ Tex in those from SLE patients (Figure 3D). Heatmap of the DEGs from cluster 16 showed that CD74 and CD7 were the most upregulated genes in SLEs compared with HDs (Figure 3E), and therefore CD74^high^CD44^high^ was used to further characterize the cluster 16. Flow cytometry assay revealed that, compared with the control group, SLE patients displayed a sharp reduction in the total CD8+ Tex (Figures S4A and 3F). To further evaluate the potential association between CD7^high^CD74^high^ T cells and SLE pathogenesis, we expanded the population of our research subjects (36 controls and 52 SLE patients, information shown in Table S4). As expected, a robust increase in CD7^high^CD74^high^ T cell proportion was detected in SLE patients, compared with the controls (Figure S4B,G).

The clinical significance of CD7^high^CD74^high^ T cells in SLE patients were further explored in the 52 SLE patients. Linear regression assays revealed that the percentage of CD7^high^CD74^high^ T cells was strongly associated with SLE‐associated autoimmune antibodies, including anti‐dsDNA antibodies (Figure S4C; *R* ^2^ = 0.5919, *p* < 0.001) and anti‐nRNP/Sm antibodies (Figure S4D; *R* ^2^ = 0.5919, *p* < 0.001), two SLE diagnostic indicators commonly used in the clinic. Receiver operating characteristic (ROC) model revealed that exhibited a superior diagnostic value, with an excellent AUC value of 0.9947 (*p* < 0.0001; Figure 3H). Moreover, high levels of CD7^high^CD74^high^ T cells were positively associated with the female gender and higher SLE activity (Table S5). These data indicate that CD7^high^CD74^high^ T cells, robustly increased in SLE patients, may be regarded as a potential diagnostic indicator in SLE.

Reduced cytolytic function of CD8+T cells, which are also known as exhausted CD8+T cells (CD8+ Tex), are believed to promote systemic autoimmunity and contribute to organ damage [21, 22]. Therefore, the expression of key proteins of CD8+T cell exhaustion (also known as immune checkpoint molecules) including programmed death receptor 1 (PD‐1), T lymphocyte immunoglobulin mucin‐3 (TIM3), and cytotoxic T lymphocyte‐associated protein 4 (CTLA‐4), in CD7^low/mid^CD74^low^ T cells and CD7^high^CD74^high^ T cells from aHDs and aSLEs, was evaluated using flow cytometry. The results showed that CD7^high^CD74^high^ T cells had much higher percentages of PD‐1^high^, TIM3^high^, and CTLA‐4^high^ cells than CD7^low/mid^CD74^low^ T cells (Figure S4E), and they also expressed much lower levels of landmark immunoactive cytokines in CD8+T cells, including IFN‐γ, IL‐2, and tumor necrosis factor (TNF)‐α (Figure S4F). Dual immunofluorescence staining also revealed that, compared with the controls, CD8+NKG7+ T cells derived from SLE patients displayed significantly higher levels of CD7 and CD74 (Figure S5A), and TdT‐mediated dUTP nick‐end labeling (TUNEL) staining revealed that the apoptotic proportion of CD8+NKG7+ T cells derived from SLE was obviously higher than that in controls (Figure S5B). Gene set enrichment analysis revealed that DEGs of cluster 16 are linked to multiple immunological and inflammatory‐related biological processes, such as leukocyte mediated immunity, cell adhesion, leukocyte activation, type II IFN response, and regulation of inflammatory (Figure S6A), enriched in multiple immunological‐related pathways, such as virus infection, antigen processing and presentation, autoimmune disease and graft‐versus‐host disease (Figure S6B,D), and associated with SLE‐related phenotypes of multiple tissue damage, such as abnormality of the kidney, abnormality of retinal morphology, abnormality of the skin, meningitis, and edema (Figure S6C,E). These data suggest that CD7^high^CD74^high^ T cells in SLE patients display exhausted features, and they are likely to contribute importantly to SLE pathogenesis.

### 2.3 SLE Patients Exhibit a Distinct m5C Landscape from the Controls

In the second stage of this study, m5C epitranscriptome sequencing was performed in CBCs from six SLE patients (P_2_) and six HCs (C_2_). Our results showed 1544 upregulated and 743 downregulated genes in patients with SLE (Figure S7A,B). Gene Ontology (GO) and Kyoto Encyclopedia of Genes and Genomes (KEGG) analyses showed that the differentially expressed genes (DEGs) were significantly associated with macromolecule catabolic processes (Figure S7C), multiple protein/enzyme binding (Figure S7D), and formation of multiple complexes (Figure S7E), and were enriched in infection, autoimmune, and cellular stress‐related pathways, such as virus infection, endoplasmic reticulum stress, and SLE (Figures S7F and S8).

**FIGURE 4:**
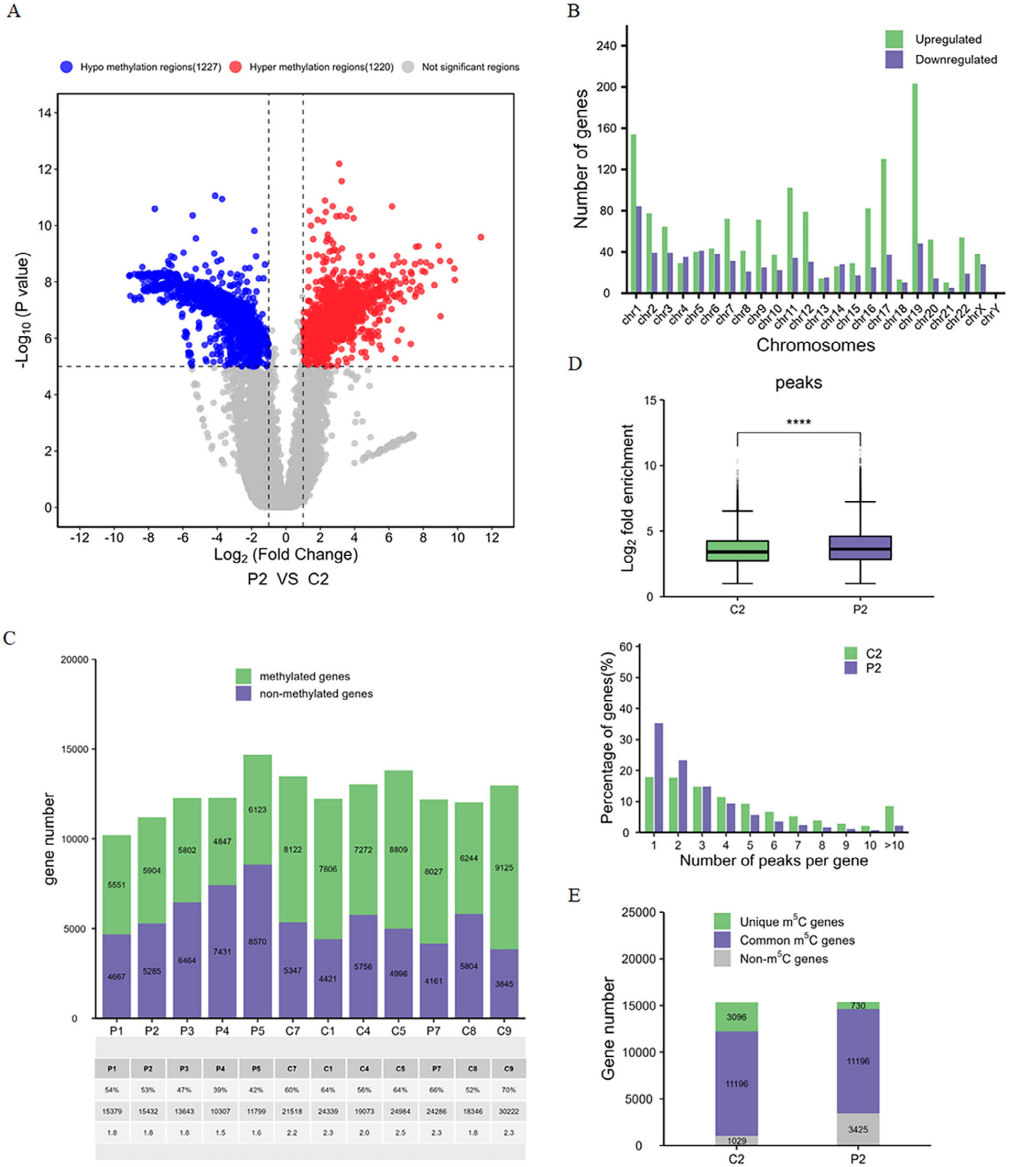
m5C‐seq analysis for the DMGs and their distributions in chromosomes and samples. m5C epitranscriptome assay was performed in six SLE patients and six controls. MACS software was applied to identify the methylation genes in each sample, diffReps software was applied for differential methylation gene (DMG) identification. Peaks located on the exon of mRNA were screened and annotated. (A) Volcano plots of the DMGs. (B) Chromosome distribution of DMGs. (C) The percentages of methylated genes and nonmethylated genes in each sample. (D) The percentages of genes with different number of m5C peaks. (E) Gene numbers of unique m5C‐modified genes, common m5C‐modified genes, and non‐m5C‐modified genes in C_2_ and P_2_ groups.

Comparison of the m5C epitranscriptome revealed that there were 1220 hyper and 1227 hypo m5C‐modified genes in SLE patients (Figure 4A), and their chromosome distribution is shown in Figure S9. It is interesting that SLE patients have many more upregulated DEGs but nearly equal numbers of hyper m5C‐modified genes to the controls (Figures 4B and S11), that is to say, high m5C levels are not equivalent to high gene expression levels. We then observed the distribution of methylated (m5C‐modified) genes in each sample and the methylated peaks in each gene. The results showed that the proportion of methylated genes in SLE patients was smaller than that in the control (Figure 4C), but there were more m5C peaks in SLE patients, since SLE patients had a large proportion of genes with single or double m5C peaks, while the controls had more genes with multiple m5C peaks (Figure 4D). GO analysis revealed that differentially methylated genes (DMGs) exhibited a high degree of similarity and coordination to the DEGs in biological processes and molecular functions: they were significantly associated with macromolecule biosynthetic processes (Figure S10A), multiple protein/enzyme binding (Figure S10B), and formation of multiple organelles (Figure S10C), and were enriched in infection, autoimmune, and cellular stress‐related pathways. KEGG analysis indicated that DMGs were enriched in pathways associated with cell behaviors, gene expression regulation, and cell signal transduction, such as endocytosis, transcriptional misregulation in cancer and insulin resistance (Figure S10D).

Intersection analysis between DEGs and DMGs indicated that most m5C genes were shared in the controls and SLE patients, while SLE patients had much less unique m5C but many more non‐m5C genes (Figure 4E). Further intersection analyses between DEGs and DMGs revealed that 15.9% of genes were upregulated and 3.1% were downregulated in genes with hyper m5C modification (Figure S11A), while in genes with hypo m5C modification, 16.4% of genes were upregulated and 2.4% were downregulated (Figure S11B). In contrast, in the upregulated genes, 9.1% of genes had hyper m5C modification and 9.8% had hypo m5C modification (Figure S11C), while 12.9% of genes had hyper m5C modification and 10.7% had hypo m5C modification in the downregulated genes (Figure S11D). These data suggest that the impact of overall changes in m5C modifications on gene expression levels can be ignored at the scale of the entire genome.

### 2.4 Conjoint Analyses of scRNA‐seq and m5C‐seq Reveal the Crucial Role of NSUN4 in Regulating CD8+T Cell Exhaustion

**FIGURE 5:**
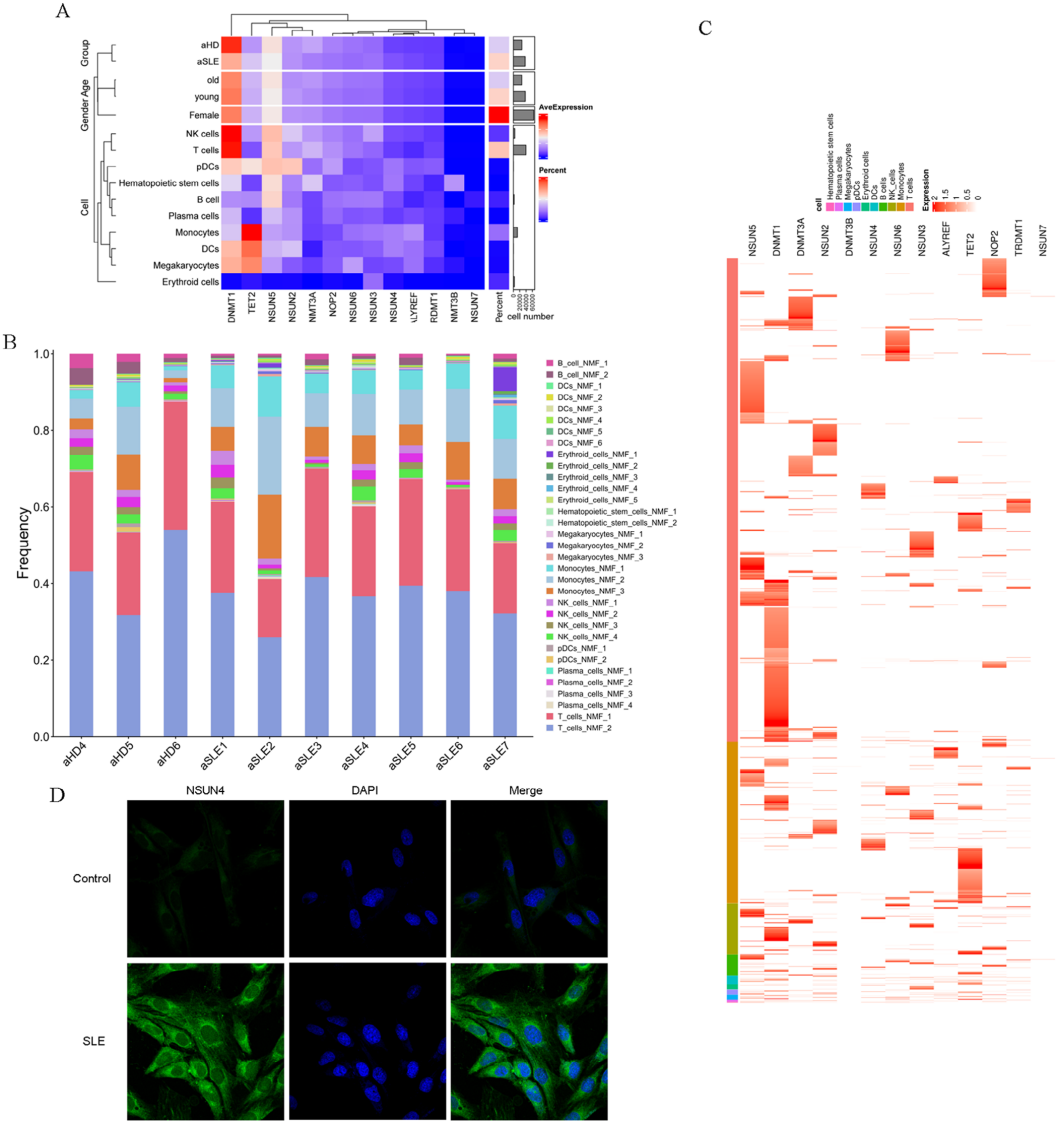
NSUN4 was screened as a key m5C regulator in SLE through conjoint analysis of scRNA‐seq and m5C‐seq. (A) Average expression of m5C regulators in the single cells based on different clinical variables containing cell types by using *z*‐score. (B) NMF clustering for the differentially expressed m5C regulators in different cell types. (C) Heatmap distribution of differentially expressed m5C regulators in different cell types. (D) Immunofluorescence staining was used to evaluate the NSUN4 levels in CD8+NKG7+ T cells from Controls and SLEs. ×400.

We conjointly analyzed the single‐cell transcriptome and m5C epitranscriptome, and the results showed that most of the significantly changed m5C regulators, including ten‐eleven translocation 2 (TET2), NOP2/Sun RNA methyltransferase 2 (NSUN2), NOP2/Sun RNA methyltransferase 3, NSUN4, tRNA aspartate methyltransferase 1, DNA methyltransferase 3β, and NOP2/Sun RNA methyltransferase 7 (NSUN7), were upregulated in SLE patients, especially m5C eraser TET2 and writer NSUN4 (Figure 5A). Non‐negative matrix factorization (NMF) was used to assess the frequencies of all cell lineages in each sample and the expression proportions of m5C regulators in each cell lineage. The results showed that TET2 displayed a much higher proportion in monocytes (Figure 5B,C), rather than T cells, and the role of TET2 in immune‐related diseases has been well characterized; therefore, we chose *NSUN4* as a candidate gene in our subsequent research on T cells. Immunofluorescence staining was used to evaluate the NSUN4 levels in CD8+NKG7+ T cells from the controls and SLEs. As expected, CD8+NKG7+ T cells derived from patients with SLE displayed an obviously higher level of NSUN4 protein (Figure 5D).

**FIGURE 6:**
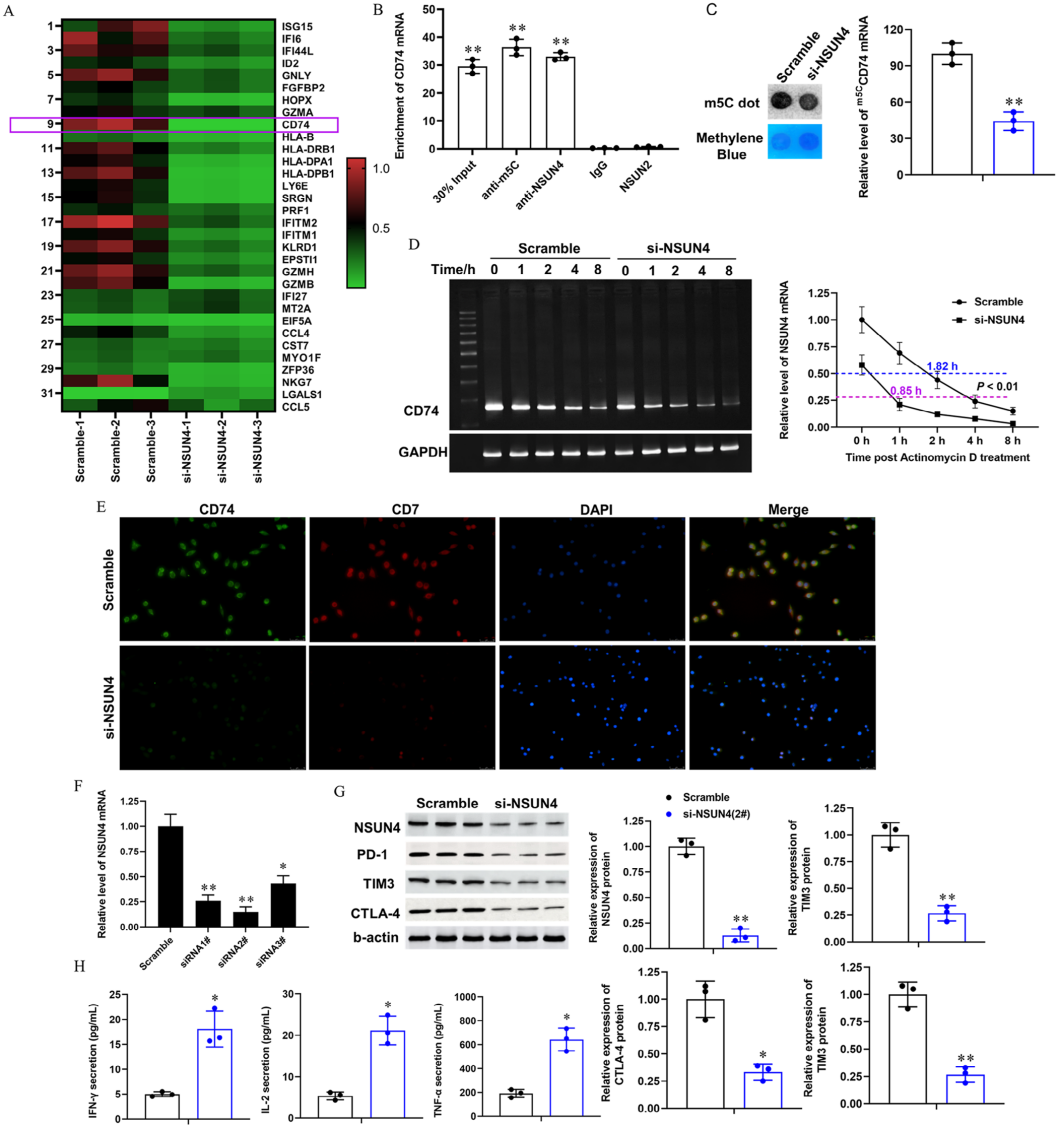
Knockdown of *NSUN4* suppresses exhaustion of CD8+ T cells in vitro. 50 nM *NSUN4* siRNAs or scrambled siRNA was transfected into cultured CD8+NKG7+ T cells by using the PEI–PBA (polyvinylimine system modified with phenylboronic acid) system. After 72 h, cells were harvested for functional evaluation. (A) The m5C high‐throughput microarray was used to evaluate the m5C levels of DEGs in cluster 16. (B) RIP and m5C antibody‐based methylated RIP (meRIP) assays were used to verify the binding of NSUN4 with *CD74* mRNA. (C) Spot hybridization and meRIP–qPCR were respectively applied to evaluate the effect of silencing NSUN4 on overall level of m5C transcripts and m5C‐modified *CD74* mRNA. (D) Actinomycin D was used to incubate CD8+NKG7+ T cells the effect of silencing NSUN4 on the stability of *CD74* mRNA. (E) Dual immunofluorescence staining was used to evaluate the CD7 and CD74 levels in CD8+NKG7+ T cells with or without NSUN4 knocked down. DAPI was used to mark the nucleus (blue). ×200. (F) Interference efficiencies of NSUN4 siRNAs detected with qPCR. (G) Western blotting was applied to detect the protein levels NSUN4 and CD8+ T dysfunctional markers, including PD‐1, TIM3, and CTLA‐4. (H) ELISA was used to detect the secretion of landmark immunoactive cytokines of CD8+T cells, including IFN‐γ, IL‐2, and TNF‐α. *N* = 3, *p < 0.05, **p < 0.01 compared with scramble.

DEGs in each cell cluster were then screened to identify genes downstream of *NSUN4*. Our results showed that there were 59 DEGs in cluster 16, with 57 upregulated and two downregulated in SLE patients (Table S2). GO and KEGG analyses showed that they were mainly associated with the structure and function of membranes, ion transport, and ribosome functions (Figure S11), and enriched in pathways related to immune, autoimmune, and immune rejection‐related disorders (Figure S12), among which *CD74* was the most upregulated. Next, *NSUN4* was knocked down in cultured CD8+NKG7+ T cells (the interference efficiencies are shown in Figure 6F,G), and a microarray‐based method for quantificationally detecting methylation changes of m5C modification was used to screen the optimal target of NSUN4. Our results showed that *CD74* was also the most m5C downregulated gene in response to *NSUN4* knockdown (Figure 6A). RNA immunoprecipitation (RIP) and meRIP assays were used to verify the regulation of NSUN4 on CD74. The results showed that *CD74* mRNA was enriched in RNA–protein complexes precipitated by m5C and NSUN4 antibodies, but not in those precipitated by IgG or NSUN2 antibody (Figure 6B). Moreover, the knockdown of *NSUN4* decreased the overall level of m5C transcripts in CD8+T cells and m5C‐modified *CD74* mRNA (Figure 6C). The most important effect of m5C in RNA processing is to make the modified RNA more stable; therefore, we then detected *CD74* mRNA stability with or without silencing *NSUN4*. The results showed that, as expected, silencing *NSUN4* significantly decreased the stability of *CD74* mRNA (Figure 6D).

Subsequently, the effect of silencing *NSUN4* on CD8+T cell exhaustion was evaluated. A dual immunofluorescence assay showed that the expression levels of CD7/CD74 were sharply reduced (Figure 6E) and the proportion of apoptotic cells was increased (Figure S14A) when *NSUN4* knocked down. Knockdown of NSUN4 also suppressed the expression of CD8+T cell exhaustion markers PD‐1, TIM3, and CTLA‐4 (Figure 6G) and improved the secretion of immunoactive cytokines IFN‐γ, IL‐2, and TNF‐α (Figure 6H) in CD8+NKG7+ T cells in vitro. These findings suggest that *NSUN4* knockdown suppresses the expression of CD74 in an m5C‐dependent manner and inhibits CD8+T cell exhaustion.

### 2.5 Knockdown of NSUN4 Suppresses Mitophagy and Improves Mitochondrial Function in CD8+T Cells

**FIGURE 7:**
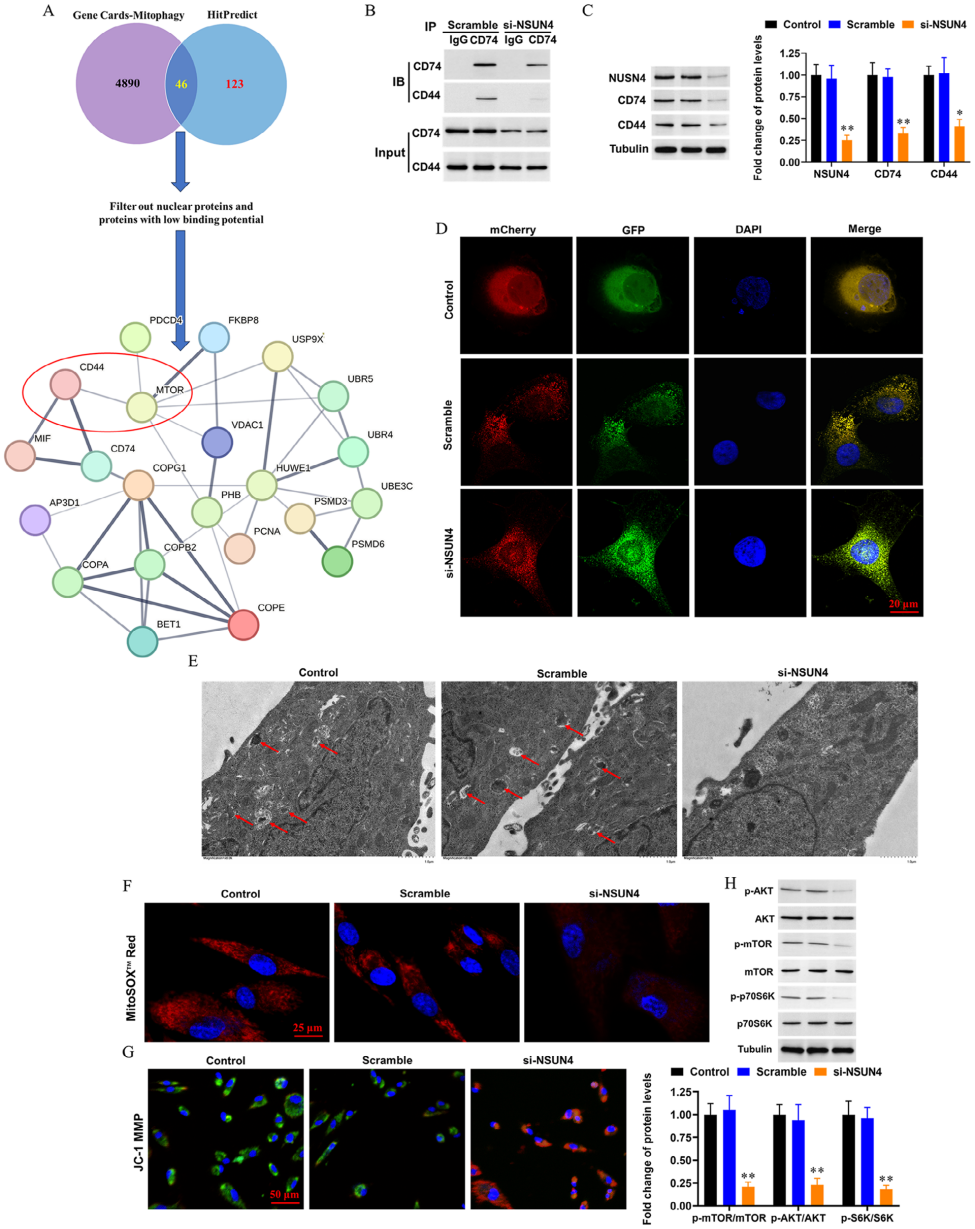
Knockdown of *NSUN4* inactivates the CD44/mTOR/S6K pathway and improves mitophagy in CD8+ T cells in vitro. (A) The screening process of the interacting proteins of CD74. (B) Coimmunoprecipitation (co‐IP) was used to verify the binding capacity of CD74 and CD44. 50 nM *NSUN4* siRNAs or scrambled siRNA was transfected into cultured CD8+NKG7+ T cells by using the PEI–PBA system. After 72 h, cells were harvested for functional evaluation. (C) Western blotting was used to detect the expression of NSUN4, CD74, and CD44. (D) Autophagic flux assay was used to evaluate the level of gross autophagy. DAPI was used to mark the nucleus (blue). ×1000. (E) Transmission electron microscopy was used to observe damaged mitochondria and mitochondrial autophagosomes in the cells. ×8000. The arrows indicate the mitochondrial autophagosomes. (F) MitoSOX^TM^ Red was used to evaluate the level of mitochondrial oxidative stress. (G) JC‐1 fluorescence probe was used to evaluate the mitochondrial membrane potential. *N* = 3, *p < 0.05, **p < 0.01 compared with control and scramble.

Furthermore, we explored the underlying mechanism by which NSUN4/CD74 regulates CD8+T cell exhaustion. Mitophagy is an important biological process that regulates the cellular energy supply and normal cellular function. Recently, mitophagy was found to be dysregulated during T‐cell exhaustion [23, 24]. CD74 has been previously reported as a key contributor to mitochondrial dysfunction in several types of metabolically active cells, such as cardiomyocytes and macrophages [25, 26]. However, its role in mitophagy‐mediated mitochondrial function during CD8+T‐cell exhaustion has not yet been reported. In this section, we comprehensively evaluated the effect of silencing *NSUN4* on mitophagy and mitochondrial dysfunction. A total of 4936 mitophagy‐related protein‐coding genes were searched from the online bioinformatics website Genecards (https://www.genecards.org/Search/Keyword?queryString=mitophagy&startPage=7&geneCategories=ProteinCoding), and they were intersected with the 169 interacting protein of CD74 from HitPredict (https://www.hitpredict.org/htp_int.php?Value=19929). After filtering out nuclear proteins and proteins with low binding potential, we obtained a protein–protein interaction network composed of CD74 and other 21 proteins (Figure 7A). CD44, an mTOR (mechanistic target of rapamycin kinase)‐related protein, was selected as a targeting protein of CD74. Co‐IP assay indicated that CD74 can directly bind to CD44 in CD8+NKG7+ T cells (Figure 7B). Western blotting results showed that knockdown of *NSUN4* had a moderate suppressive effect on CD44 protein (Figure 7C). Autophagic flux assay demonstrated that silencing NSUN4 markedly suppressed gross autophagy of CD8+T cells (Figure 7D), and transmission electron microscopy showed an obvious increase in abnormal mitochondria and mitochondrial autophagosomes in *NSUN4*‐silenced CD8+T cells (Figure 7E). MitoSOX Red and JC‐1 fluorescence probe staining showed that the level of mitochondrial oxidative stress and mitochondrial membrane potential were obviously improved by silence of *NSUN4* (Figure 7F,G). Consistent with the changes in subcellular morphology, TUNEL staining showed that knockdown of *NSUN4* obviously decreased CD8+T cell apoptosis, and Western blotting assays showed that the levels of mitochondrial fission markers dynein‐related protein 1 and mitochondrial fission 1 were decreased, the levels of mitochondrial fusion marker mitofusin and mitophagy markers PINK1 (PTEN‐induced putative kinase 1) and Parkin were improved, and adenosine triphosphate (ATP) production, the most important indicator of mitochondrial function, was also improved by the knockdown of *NSUN4* (Figure S14B,C). It was revealed that mTOR activates mitophagy through promoting the phosphorylation of the ribosomal protein S6 kinase (p70S6K). Therefore, Western blotting was used to detect the levels of phosphorylation of p70S6K and protein kinase B (AKT). As expected, knockdown of *NSUN4* suppressed phosphorylation of mTOR, p70S6K and AKT in CD8+NKG7+ T cells (Figure 7H). These data demonstrate that the knockdown of NSUN4 improves mitophagy and mitochondrial function in CD8+T cells, which may be related to the CD74/CD44/mTOR/S6K pathway.

### 2.6 Nanoparticle‐Delivered siRNA Against NSUN4 Alleviates SLE in Vivo

**FIGURE 8:**
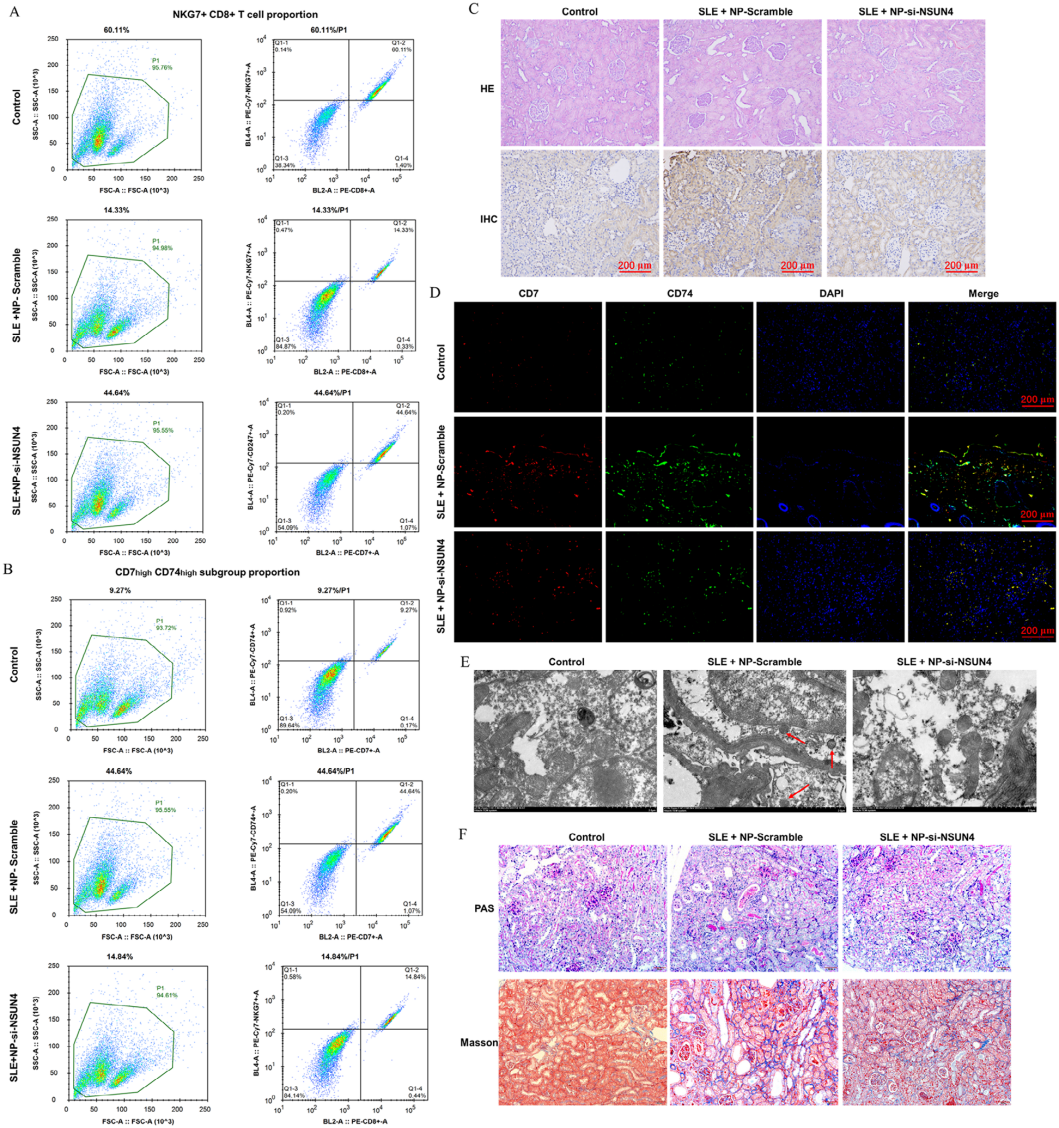
Knockdown of *Nsun4* alleviates CD7^high^CD74^high^ CD8+T cell infiltration, mitophagy, and kidney tissue damage in SLE mice. Spontaneous lupus MRL/lpr mice applied to investigate the role of *Nsun4* in SLE progression. Liposome–protamine–hyaluronic acid (LPH) nanoparticle (NP)‐delivered siRNA against *Nsun4* was administrated into MRL/lpr mice by the tail vein injection and the NP‐scramble was used as a negative control. Blood was collected from the controls and SLE mice, and CD8+ T subgroups were segregated and counted with flow cytometry. The proportions of (A) NKG7+CD8+ T cells and (B) CD7^high^CD74^high^ CD8+T cells in each group. (C) After blood collection, the kidney tissues were collected, spliced and stained with HE and immunohistochemistry to observe the tissue morphology and NSUN4 expression. (D) Dual immunofluorescence was used to observe the infiltration of CD7^high^CD74^high^ T cells in the kidney. (E) Transmission electron microscopy was used to observe damaged mitochondria and mitochondrial autophagosomes in the kidney. (F) PAS staining and Masson staining were to observe the integrity of basement membrane and degree of fibrosis of the kidney tissue. *N* = 6.

Spontaneous lupus MRL/lpr mice were used to investigate the role of Nsun4 in SLE progression, with normal C57L/6 mice as a control. Nanoparticle‐delivered siRNA against *Nsun4* was administrated into MRL/lpr mice by tail vein injection and scrambled siRNA was used as a negative control. Blood was collected from the control and SLE mice, and CD8+ T cell subgroups were segregated and counted using flow cytometry. Our results showed that MRL/lpr mice displayed a sharp reduction in NKG7+CD8+ T cell proportion, while knockdown of *Nsun4* markedly rescued the reduction of NKG7+CD8+ T proportion (Figure 8A). In contrast, the CD7^high^CD74^high^ T cell proportion was robustly increased in MRL/lpr mice, which was predominantly reduced by the knockdown of *Nsun4* (Figure 8B). Skin and kidney tissues were stained with HE and IHC. The results showed that compared with normal mice, MRL/lpr mice displayed a damaged tissue structure, disrupted cell integrity, increased infiltration of inflammatory cells, and even increased necrotic areas in the skin and kidney, accompanied by an obvious increase in Nsun4 expression, whereas nanoparticle‐delivered NSUN4 siRNA largely rescued the tissue damage and fibrosis and diminished the Nsun4 level increase (Figures 8C,F and S15A). Consistent with the proportions in the blood, dual immunofluorescence assay also revealed that CD7^high^CD74^high^ T cells were hardly observed in the skin and kidney of normal mice, but were obviously observed in MRL/lpr mice, but noticeably declined in MRL/lpr mice with NSUN4 knockdown (Figures S15B and 8D). Transmission electron microscopy was used to observe damaged mitochondria and mitochondrial autophagosomes in the kidney. The results showed that nanoparticle‐delivered *Nsun4* siRNA obviously improved the mitochondrial morphology and reduced mitochondrial autophagosomes in the CD8+ T cells in the kidney (Figure 8E). Furthermore, kidney function and serum autoimmune antibody levels were evaluated in each group. Our data showed that MRL/lpr mice displayed significantly lower spleen and kidney weight indices and higher levels of serum dsDNA antibodies, nRNP/Sm antibodies, urine albumin/creatinine ratio, and serum cystatin C (Figure S16A–F), suggesting a damaged kidney function and high level of autoimmunity. Also, knockdown of NSUN4 largely rescued these indexes in SLE mice (Figure S16A–F). To further verify the therapeutic effect of *Nsun4* knockdown, nanoparticle‐delivered *Nsun4* siRNA was administrated into pristane‐induced SLE mice by tail vein injection and scrambled siRNA was used as a negative control. Inconsistent with the results from spontaneous SLE mice above, nanoparticle‐delivered *Nsun4* siRNA rescued the tissue damage and fibrosis and diminished the Nsun4 level increase (Figure S17A). Also, nanoparticle‐delivered *Nsun4* siRNA largely rescued the decreased spleen and kidney weight indices, higher levels of serum dsDNA antibodies, and increased urine albumin/creatinine ratio in pristane‐induced SLE mice (Figure S17B–E). One more important thing is that, compared with male pristane‐induced SLE mice, the above indicators of female pristane‐induced SLE mice displayed more deviations from normal values; while nanoparticle‐delivered *Nsun4* siRNA has nearly the same therapeutic effect on male female pristane‐induced SLE mice (Figure S17B–E). These findings indicate that knockdown of *Nsun4* by nanoparticle‐delivered siRNA alleviated SLE in both spontaneous SLE mice and pristane‐induced SLE mice in vivo.

## 3 Discussion

In this study, we first explored the heterogeneities of blood cells and their associations with SLE pathogenesis by performing single‐cell transcriptome sequencing of CBCs from SLE patients and healthy donors. In the cell composition analysis, we found an interesting phenomenon that cluster 10, which represented total B cells, was sharply reduced in the SLE blood. It is widely acknowledged that B cell dysfunction plays a significant role in the pathogenesis of SLE. SLE patients usually have an increased number of mature B cells and more activated B cells that secrete autoimmune antibodies, but there are exceptions in some cases, such as when the patients are in an active phase, the immunity of the patients is severely declined, and the patients are receiving treatment or undergoing side effects of medication [27, 28, 29]. Here, the SLE patients enrolled in the scRNA‐seq were all in an active phase; therefore, it is not difficult to understand that their B cells were reduced. Another noteworthy change in cell composition is that all clusters belonging to monocytes were expanded, suggesting the potential contribution of monocytes to SLE pathogenesis. Moreover, most subgroups of T cells were reduced, whereas cluster 16 was robustly expanded, in which we identified a CD7^high^CD74^high^ CD8+T cell subset that displayed exhausted features and might be associated with the pathogenesis of SLE. The role of CD8+T cells in SLE is worth discussing. Some researchers believe that the high cytotoxicity of the CD8+T cell contributes to SLE pathogenesis. Xiong et al. [22] identified a highly cytotoxic CD8+ T cell subset, defined as the CD161‐CD8+ T_EMRA_ cell subpopulation, which was expanded in patients with SLE. Conversely, dysfunction, loss of CD8, or death of CD8+T cells, weakens them in the control of autoimmunity and risk of infections, and functional defects of CD8+T cells also contribute to SLE pathogenesis [4, 21]. A typical example is that Buang et al. [5] reported that in the “SLE‐like” conditions that CD8+T cells from healthy volunteers were exposed to type I IFN and TCR stimulation, CD8+T cell NAD+ consumption was increased, cell viability was reduced, and cell death was promoted. In this study, the CD7^high^CD74^high^ CD8+T cell subset, identified in cluster 16, displayed exhausted features, including high levels of PD‐1, TIM3, and CTLA‐4, lower levels of IFN‐γ, IL‐2, and TNF‐α and a higher proportion of apoptotic cells. In the subsequent research in an expanded population of subjects, we demonstrated that CD7^high^CD74^high^ CD8+T cells were indeed significantly expanded in SLE patients compared with the controls, suggesting that exhaustion of CD8+T cells might contribute to SLE pathogenesis. ROC model revealed that exhibited a superior diagnostic value, with an excellent AUC value of 0.9947, and high levels of CD7^high^CD74^high^ T cells were positively associated with higher SLE activity, suggesting that CD7^high^CD74^high^ T cells may be regarded as a potential diagnostic indicator in SLE.

In clusters belonging to T cells, clusters 0, 2, 3, 4, 7, 8, 9, 11, 12, 18, and 24 were reduced, whereas clusters 16 and 25 were expanded. Since the number of cluster25 was extremely small (less than 1/2000 of the total cell number in aSLEs, and less than 1/5000 of the total cell number in aHDs), we neglected their potential influences. As for cluster 16, previous studies have revealed that CD3D, CD3E, CD8A, GZMA, GZMH, and NKG7 can be used to characterize them: CD3+CD8+ cells are a typical description of CD8+T cells, and GZMA, GZMH, and NKG7 are markers for subgroups of CD8+T or NK cells [30, 31, 32]. Previous studies have revealed a CD3+CD8+GZMH+/NKG7+ subgroup associated with SLE [7, 33]. However, in our validation of the expanded subject population, we found that the CD7^high^CD74^high^ subset displayed a robust increase in the gate of CD8+NKG7+ cells but a less drastic increase in the gate of CD8+GZHM+ cells in SLE patients. The most important reason we think is that, compared with GZMH, NKG7 is closer to CD8+ T cells, as a number of reports have indicated that NKG7 is required for CD8+ T immune activity in antitumor cytotoxicity, cancer immunotherapy, and anti‐inflammation [34, 35, 36].

When we tried to define the markers of the robust increased subcluster in aSLEs, there were a few other choices apart from CD7 and CD74 (Table S5). After a comprehensive consideration of their roles in immune‐related diseases, we decided to adopt them. CD7 is now famous for its wide application in the immunotherapy of hematological malignancies. Naturally occurring CD7‐T and genetically modified CD7‐targeting allogeneic CAR‐T cells both displayed an ideal therapeutic effect on hematological malignancies especially acute T‐cell lymphcarcinoma [37, 38, 39]. As early as the 1990s, researchers realized that CD7 might contribute to the pathogenesis of rheumatic diseases, and the effect of monoclonal antibody to CD7 on rheumatic diseases was also evaluated in the clinical setting [40]. Research in children with SLE has shown that CD7+ lymphocytes expressing high levels of P‐gp1 are positively correlated with disease activity. Therefore, in this study, we selected CD7 as a marker protein of the robust subcluster in SLE cluster 16 from the potential marker proteins [41].

CD74 is a chaperone of the major histocompatibility complex‐II, which has been demonstrated to play important roles in many inflammatory and autoimmune diseases, such as kidney diseases [42], liver fibrosis [43], cardiovascular diseases [44], cancers [45, 46, 47], infections [48], autoimmune encephalomyelitis [49], spondyloarthritis [50], and SLE [51, 52]. CD74 functions as a transcription regulator receptor that regulates the activity of multiple immune cells, including B cells [53], macrophages [54], CD4+ T cells [55], and hematopoietic stem cells [56]. Anti‐CD74 IgG and IgA antibodies were markedly increased in spondyloarthritis patients, displaying a high diagnostic value in axial spondyloarthritis and positive association with disease activity [57]. The blockade of CD74 expression and its partner macrophage migration inhibitory factor has been applied as a targeted therapy for autoimmune encephalomyelitis and rheumatoid arthritis [49, 52]. In SLE studies, CD74 was identified as a marker of exhausted regulatory CD4+T cell subset that contribute to SLE pathogenesis and showed therapeutic potential when blocked or knocked out in SLE animal models [10, 58, 59]. However, it was not clear what the role of CD74 in SLE CD8+T cells. In our study, we found that CD74 was the most upregulated gene in the identified exhausted CD8+T cell subcluster and played a suppressive role in CD8+T cell function and survival and that deregulation of CD74 by interfering with the expression of NSUN4 decreased tissue infiltration and the proportion of circulating CD7^high^CD74^high^ CD8+T cells, serum contents of autoimmune antibodies, and tissue damage of the skin and kidney in SLE mice.

As the “power station” of cells, mitochondria supply the entire cell with energy and ensure normal operation. Dysregulation of mitochondrial dynamics, including mitochondrial transport, fusion, division, and differentiation, is the basic pathogenesis of almost all human diseases. Recently, disturbed mitochondrial dynamics were reported to be associated with CD8+T‐cell exhaustion [23, 24, 60]. Mitophagy is a specific type of autophagy that focuses on the clearance and repair of damaged mitochondria to maintain the integrity of mitochondrial network function and cellular homeostasis. Mitophagy has been involved in multiple diseases related to inflammation and immunity, and improvement of mitochondrial function by modulating mitophagy is acknowledged as a promising therapeutic strategy for SLE. For example, the cytoskeleton protein nestin was found upregulated in the podocytes of MRL/lpr SLE mice, accompanied with enhanced mitophagy and oxidative stress; knockdown of nestin was able to suppress mitophagy and oxidative stress, reduce podocyte damage and alleviate nephritis in vivo [61]. Perl et al. [62, 63] also reported that dysfunction of mitochondria was observed in peripheral blood lymphocytes of SLE patients and MRL/lpr mice, and that improving mitophagy by deletion of Rab4A or inhibition of the mTOR signaling facilitates the expands of CD4+T cells and reduces CD8+ T cells, thereby enhancing B cell activation, plasma cell development, antinuclear and antiphospholipid autoantibody production, and glomerulonephritis. In SLE and other autoimmune diseases, such as rheumatoid arthritis, dysregulation of mitophagy promotes excessive oxidative stress and may lead to overactivation of the cellular immune system and the characteristic development of autoantibodies  which contribute to sensitivity of T cells for necrosis and inflammation in patients [64, 65]. Mitophagy was also revealed to be dysregulated in CD8+T cell exhaustion, and improvement in mitophagy was demonstrated to inhibit T cell exhaustion and enhance their immune activity [25, 26]. In this study, we reported that knockdown of NSUN4 had a protective effect against CD8+T cell exhaustion, which was associated with m5C‐mediated downregulation of CD74 expression and improvement of mitophagy. CD74 has been previously reported as a key regulator of mitochondrial dysfunction in several types of metabolically active cells, such as cardiomyocytes and macrophages. However, its role in mitophagy‐mediated mitochondrial function during CD8+T cell exhaustion has not yet been reported. In this study, we screened the target protein using the online bioinformatics website Genecards and the online protein–protein interaction prediction tool HitPredict. After filtering out nuclear proteins and proteins with low binding potential, we obtained a small protein–protein interaction network and selected CD44 as the candidate protein, for its important role in the activation of mTOR/S6K‐mediated mitophagy. Co‐IP and further functional experiments indicated that downregulation of CD74 by NSUN4 knockdown had a suppressive effect on the mTOR/S6K pathway and mitophagy in primary SLE NKG7+CD8+T cells.

m5C is one of the major types of RNA modification that has been involved in many major human diseases. Compared with other major diseases, our knowledge of the m5C landscape in SLE is relatively limited [19, 66]. In our study, m5C epitranscriptome sequencing of CBCs from SLE patients and HCs showed that there were 1544 upregulated and 743 downregulated genes in SLE patients, while there were 1220 hyper and 1227 hypo DMGs in SLE patients. It is interesting that SLE patients have many more upregulated DEGs but nearly equal numbers of hyper methylated genes to the controls, suggesting high m5C levels are not equivalent to high expression levels in SLE patients. Moreover, intersection analysis between DEGs and DMGs indicated that 15.9% of genes were upregulated and 3.1% were downregulated in genes with hyper m5C modification, while in genes with hypo m5C modification, 16.4% of genes were upregulated and 2.4% were downregulated; in contrast, in the upregulated genes, there was 9.1% of genes with hyper m5C modification and 9.8% with hypo m5C modification, whereas there was 12.9% of genes with hyper m5C modification and 10.7% with hypo m5C modification in the downregulated genes. These data further indicated that hyper m5C modification does not cause significant upregulation of genes in the scope of the full transcriptome, and vice versa as hypo m5C. Interestingly, although there are many discrepancies between DEGs and DMGs, functional annotation results of GO and KEGG indicated that the biological processes and pathways in which they participate are highly similar or collaborative: DEGs and DMGs were both enriched in immune‐related pathways. Elevated m5C modification mainly promotes the stability, translation, transcription, and nuclear export of RNAs [67, 68], while facilitating degradation in rare cases. Our findings, to some extent, overturned our understanding of the effect of m5C modification on gene expression.

By conjoint analysis of the single‐cell transcriptome and m5C epitranscriptome, we showed that most of the significantly changed m5C regulators were upregulated in SLE patients, especially the m5C eraser TET2 and the writer NSUN4. Finally, we abandoned TET2 in subsequent research on T cells. NMF analysis showed that TET2 displayed a much higher proportion of monocytes than T cells [69, 70]. On the other hand, the role of TET2 in immune‐related diseases have been well characterized, but its role in SLE is controversial as presented in limited studies. A study showed that the m5C eraser TET2 was significantly upregulated and associated with the levels of biomarkers indicative of SLE disease activity [71]. However, another study indicated that downregulation of TET2 is associated with the risk of SLE and might contribute to SLE progression [72]. NSUN4 is a member of the NOP2/Sun RNA methyltransferases, which were originally found to regulate rRNA methylation [73]. Recently, NSUN4 was also identified as an m5C writer for mRNA molecules, similar to other family numbers NSUN2, NSUN5, NSUN6, and NSUN7 [74, 75, 76, 77]. In our study, NSUN4 was screened as a potential regulator through conjoint analysis of the single‐cell transcriptome and m5C epitranscriptome in SLE patients, which was significantly upregulated in the CD7^high^CD74^high^ T cell subcluster and positively regulated CD74 mRNA stability and CD8+T cell exhaustion by regulating mitophagy in vitro. Moreover, knockdown of NSUN4 with nanoparticle‐delivered siRNA decreased the generation and infiltration of CD7^high^CD74^high^ CD8+T cells, serum levels of autoimmune antibodies, and skin and kidney damage in SLE mice. So far, the role of NSUN4, even most other NSUNs, in SLE has not been precisely determined. To our knowledge, this is the first report to reveal the role of NSUN4 in the pathogenesis of SLE.

Although we identified an exhausted CD8+T cell subgroup in SLE patients and revealed the role of NSUN4 and its mechanism in regulating the function of this subgroup, this study has some limitations. First, apart from T cell clusters, monocytes and B cells also exhibit drastic changes in the numbers. There may be other subgroups of monocytes or B cells associated with SLE pathogenesis, which is worth further exploration in the future. Second, the role of NSUN4 and other m5C regulators requires further exploration. In the future, we will conduct in‐depth research on the therapeutic effects of NSUN4 in SLE patients.

In conclusion, we explored the heterogeneity of blood cells and their association with SLE pathogenesis by performing single‐cell transcriptome sequencing on CBCs from SLE patients and healthy donors, and we first identified an exhausted CD7^high^CD74^high^ CD8+T cell subset that contributed to SLE pathogenesis. Moreover, we determined the m5C landscape of SLE patients and proposed the crucial role of NSUN4 in regulating CD74 expression, CD74‐mediated dysfunction of mitophagy and CD8+T cell exhaustion, and uncovered the NSUN4/CD74 axis as a potential target for SLE. Furthermore, we demonstrated that targeting NSUN4 with nanoparticle‐delivered siRNA is a promising treatment strategy for SLE patients.

## 4 Methods

### 4.1 Study Design and Data Collection

ScRNA sequencing was performed on CBCs from 13 adult SLE patients (aSLEs, aged 24–55 years, four males and nine females) and 11 healthy donors (aHDs, aged 25–58 years, four males and seven females) to explore the heterogeneity of SLE blood cells. We generated 149,513 single‐cell transcriptomes of healthy and SLE CBCs, clustered the cells using distinct markers and identified specific cell subsets associated with SLE pathogenesis. Next, m5C mRNA sequencing was performed in CBCs from six adult female SLE patients (P_2_, aged 22–39 years) and six adult female HCs (C_2_, aged 24–43 years), and DEGs and DMGs were screened and annotated to determine m5C landscape of SLE patients. Intersection analysis between DEGs and DMGs was performed to evaluate the global effect of m5C‐modification on gene expression. Conjoint analysis of scRNA‐seq and m5C‐seq was performed on m5C‐associated key regulators in SLE pathogenesis.

### 4.2 Ethical Approval

This study was approved by the Ethics Committee of Xi'an JiaoTong University (XJTU‐2022018). All patients and controls involved in this study provided informed consent.

### 4.3 Single‐Cell RNA‐seq Library Preparation and Sequencing

Peripheral blood samples were collected from SLE patients and healthy donors at the Second Affiliated Hospital of Xi'an JiaoTong University. Single‐cell suspensions were prepared as described in the 10X Genomics protocol.

### 4.4 Cell Clustering, Sample Comparative Analysis, and Subcluster Characterizing

We used the R package cellda's decontX function to remove contaminating environmental RNA and scrublet (0.2.1) software to remove duplex cells [78]. Dead or dying cells have large amounts of mitochondrial contamination. Unless necessary (e.g., cancer cells or myocardial cells, where the mitochondrial ratio is naturally high), we defined cells with a mitochondrial ratio exceeding 0.2 as samples with poor mitochondrial count quality to evaluate the number of UMIs and the number of genes detected in each cell. The Seurat R packages (v 4.2.0) [79], including Seurat NormalizeData, FindVariableFeatures, and ScaleData, were used for low‐quality cell filtration, standardization, data merging, PCA linear clustering (top 30 PC), UMAP nonlinear clustering, t‐SNE dimensionality reduction clustering, and marker gene (logfc. threshold = 0.25), and a conservative marker gene (logfc. threshold = 0.1) calculation.

Sample comparative analysis was used to identify the cell populations present in both samples, obtain conserved marker genes or clustered marker genes from two sample cells, and identify differential genes in a cluster under two different treatment conditions. Then, the SingleR package (v0 1.10.0) was supplemented by the CellMarker2.0 database and the cell marker genes for cell type identification [80, 81]. The Cluster Profiler R package (v3.18.1) was used for GO and KEGG functional enrichment analyses of the marker genes and conserved marker genes [82].

Using the FindMarkers function, we performed differential gene expression analysis on samples from the same cell group under different conditions (logfc. threshold = 0.25, min. pct = 0.1), and clusterProfiler was used to perform GO and KEGG functional enrichment analyses of the DEGs. Using the R package Monocle3, we calculated the root nodes of each cell group, selected all the root nodes for trajectory inference, and selected a single root node for cell trajectory inference [83].

### 4.5 Cell–Cell Communication and Pseudotime Analyses

Cell communication analysis was conducted using the R package Cellchat under each condition, and the cell communication statuses under the two conditions were compared [84]. Using the R package Monocle3 based on UMAP dimensionality reduction, we performed a quasi‐temporal analysis starting from the root nodes of all clusters (identified by computer programs).

### 4.6 Screening the DEGs Between HCs and SLE Patients for Each Cell Type

We removed genes in the log‐normalized gene expression matrix (after SAVER imputation) with a maximum expression value of less than 1 across all cells, and then converted the expression to a standardized *z*‐score across cells. For each cell subtype, the DEGs were screened by three thresholds: (1) *p* value < 0.01 (Mann–Whitney *U* test), FDR < 0.05 (Bonferroni correction), (2) average *z*‐score of cells from HCs ðAZnÞ > 0.1 or average *z*‐score of cells from SLE patients ðAZpÞ > 0.1, and (3) difference between AZn and AZp > 0.4.

### 4.7 Identifying the Signature Genes for Cell Clusters

We performed differential expression analysis (log2 fold change>1, *p* value < 0.05 (*t*‐test) and FDR < 0.5 (Bonferroni correction)) for each cell cluster versus all other cell clusters. The specific genes of cluster 16 were identified by differential expression analysis (log2 fold change > 0.2 and Mann–Whitney *U* test *p* value < 0.01).

### 4.8 Evaluation of the Function of CD7^high^CD74^high^ T Cells

CD8+ T cells were purified from PBMCs of controls and SLE patients using a CD3+ CD8+ T Cell Isolation Kit (NoVo Biotech, Beijing, China; #130‐096‐495) according to the manufacturer's protocols. NKG7 (E6S2A) antibody was applied in Cell Signaling Technology (Danvers, USA; #84835) flow cytometry sorting for NKG7+ CD8+ T cells. Then, anti‐CD7 (Abcam, UK; ab230834) and anti‐CD74 (Abcam; ab108393) were used to sort the CD7^high^CD74^high^ and CD7^low/mid^CD74^low^ subgroups in NKG7+ CD8+ T cells. Finally, antibodies against PD‐1 (Abcam; ab309361), CTLA‐4 (Abcam; ab231949), TIM3 (Abcam; ab47997), IFN‐γ (Abcam; ab224197), IL‐2 (Abcam; ab278101), and TNF‐α (Abcam; ab255275) were used for flow cytometry analysis for their expression in the CD7^high^CD74^high^ and CD7^low/mid^CD74^low^ subgroups.

### 4.9 m5C‐seq Library Preparation and Sequencing

RNA high‐throughput sequencing and m5C mRNA sequencing services were provided by the Shanghai Yunxu Biotechnology Co., Ltd (Shanghai, China). The GenSeq rRNA Removal Kit (GenSeq, Inc.) was used to remove ribosomal RNA (rRNA) from the sample. After removing rRNA, the sample was passed through GenSeq The Low Input RNA Library Prep Kit (GenSeq, Inc.) is used to construct a sequencing library according to the manufacturer's instructions.

The RNA m5C methylation sequencing service was provided by the Shanghai Yunxu Biotechnology Co., Ltd. Using R package ggpubr, circular plots were drawn using the R package ggpubr to evaluate the intersection of differentially m5C modified and DEGs.

### 4.10 Conjoint Analysis on the Results of scRNA‐seq and m5C‐seq

The AverageExpression function of the R package Seurat was used to calculate the mean expression data of the m5C regulator genes after standardization in different cell populations and phenotypic groups. The gene expression clustering heatmap was plotted using the R package ComplexHetmap [85]. Standardized expression data of the m5C regulator genes were extracted, and after discarding cells with all 0 expression values, ComplexHetmap was used to draw a gene expression clustering heatmap with proportions in different populations. These cells were used for NMF clustering to obtain m5C‐related subtypes. Using the development version of the NMF R package (https://github.com/renozao/NMF/tree/devel), setting the random seed to 666 and Nrun to 5, NMF clustering was performed separately for cell type, and the optimal number of clusters was selected based on the topological score. The number of cells in the NMF cluster for each cell type was counted, and ggplot2 was used to plot a frequency stacking column.

### 4.11 CD8+NKG7+ T Cell Culture and siRNA Transfection

Isolated CD8+NKG7+ T cells were resuspended in RPMI‐1640 medium, supplemented with 10% fetal bovine serum, 200 mM glutamate, and 50 ng/mL IL‐15, at a density of 1 × 10^6^/mL. 50 nM NSUN4 siRNA or scrambled siRNA was transfected using the polyethylenimine–phenylboronic acid (PEI–PBA) system as previously described [86].

### 4.12 Microarray Assay for m5C Modified Genes

After transfection for 72 h, the cells were collected and total RNA was extracted using the MagMAX FFPE DNA/RNA Ultra Kit (Thermo Fisher Tech). The m5C modification microarray high‐throughput detection service was provided by Shanghai Yunxu Biotechnology Co., Ltd. The gene expression clustering heatmap was plotted using R package ComplexHetmap.

### 4.13 Immunofluorescence and TUNEL Assays

Cells at the logarithmic growth stage were washed twice with PBS and centrifuged at 1000 rpm for 5 min. Cell slides were prepared using a cell centrifugation spreader (AF26‐C; Novo Biotech). Alexa Fluor 488 anti‐CD7 antibody (1:100, ab199022), anti‐CD74 antibody (1:100, ab223350), and CY357 labeled NSUN4 protein antibody (1:50, TF19479R; JingFeng Biotechnology, Shanghai, China) were used in the immunofluorescence assay for CD7, CD74, and NSUN4 according to the manufacturer's protocols. An Elabscience One step TUNEL in situ apoptosis detection kit (Elabscience Biotechnology Co., Ltd, Wuhan, China) was used to evaluate cell apoptosis. DAPI (#62247; Thermo Scientific Tech) was used to stain the nucleus.

### 4.14 Real‐Time Quantitative Polymerase Chain Reaction

Total RNA was extracted from T cells using MagMAX RNA Extraction Reagent (Thermo Fisher Tech). Reverse transcription was performed using a PrimeScript RT Reagent Kit (Takara Biotechnology, Dalian, China). Real‐time PCR analysis was conducted using SYBR Premix Ex Taq II (Takara) on an ABI 7500 Real‐Time PCR System (Applied Biosystems, Foster City, CA) under the following conditions: 95°C for 1 min, followed by 30 cycles at 95°C for 20 s, 56°C for 10 s, and 72°C for 15 s. Primers (Table S3) used in this study were synthesized by Sangon Biotech (Shanghai, China). Relative expression levels were normalized using the 2^−ΔΔCt^ method, with 18 RNA as a loading control.

### 4.15 Evaluation of Gross Autophagy and Mitophagy

Gross autophagy was evaluated using autophagic flux according to a previous study [87]. Mitophagy was visualized using the cell‐permeant green‐fluorescent mitochondria dye MitoTracker Green and the red‐fluorescent lysosome dye Lysotracker Red, according to the manufacturer's instructions [88]. Mitochondrial morphology and mitoautosomes were observed using transmission electron microscopy according to a previous report [89]. Mitochondrial oxidative stress level and mitochondrial membrane potential were respectively detected with a MitoSOX Red mitochondrial superoxide indicator (Invitrogen) and JC‐1 Mitochondrial Potential Sensor (Invitrogen) according to the manufacturer's instructions.

### 4.16 Preparation of Liposome–Protamine–Hyaluronic Acid Nanoparticle

Liposome–protamine–hyaluronic acid nanoparticles (LPH‐NPs) were prepared using a previously reported stepwise self‐assembly method [90]. LPH cores were prepared by mixing 140 µL of solution A (glucose resolution of 36 µg protamine) and 140 µL of solution B (5% glucose of 24 µg HA and 24 µg siRNA) at RT for 10 min. DOTAP/Chol liposome (60 µL) were dropped into the LPH cores and PEGylated with 30 µL of DSPEPEG (10 mg/mL) and 30 µL of DSPE–PEG–AA (10 mg/mL) at 50°C for 15 min. Scrambled siRNA: 5′‐TTCTCCGAACGTGTCACGTTT‐3′; NSUN4 siRNA: 5′‐GCTGGTAATACCAAACCTCAT‐3′.

### 4.17 SLE Mouse Models, NSUN4 Knockdown, and Measurements in Vivo

Sixty spontaneous lupus MRL/lpr mice and thirty normal C57L/6 mice (female, 8 weeks old, weighing 18–21 g) were purchased from the Experimental Animal Research Institute of Sichuan Provincial People's Hospital (Chengdu, China). The mice were raised in an independent ventilation cage in an SPF environment at (25 ±  2)°C and 50–70% relative humidity. After 2 weeks of acclimatization, MRL/lpr mice were administered 200 µL of nanoparticle‐delivered NSUN4 siRNA (NP‐NSUN4 siRNA) or NP‐scramble by tail vein injection. The mice were euthanized by cervical dislocation after 3 weeks. Induced SLE model was established with the intraperitoneal injection of 0.5 mL of pristane (Sigma–Aldrich; P9622–10×1ML) in SPF SJL/J mice (8 weeks old, weighing 17–24 g, half male and half female) according to a previous report [91]. Blood was collected from the eyeballs with anticoagulation, and applied to CD8+ T subgroup separation. The other part of the blood was centrifuged, and the serum was collected for detection of anti‐dsDNA antibodies, anti‐nRNP/Sm antibodies, and cystatin C with corresponding ELISA kits (Sigma). After blood collection, skin and kidney tissues were collected, frozen, or fixed in 4% paraformaldehyde solution. And HE staining, immunohistochemistry, and dual immunofluorescence, Masson staining and periodic acid Schiff (PAS) staining were used to observe tissue morphology, NSUN4 expression level, and infiltration of CD7^high^CD74^high^ T cells.

### 4.18 Statistical Analysis

Standard tests included Student's *t*‐test, Wilcoxon ranksum test, Kruskal–Wallis test, and Chi‐square test for differences in continuous target or category variables in these cell subgroups. Routine statistical analyses of the present study were performed in R 4.0 or SPSS 25.0, and a two‐sided *p* value below 0.05 was considered statistically significant.

## Author Contributions

BC. R. designed the study, prepared the m5C epitranscriptome analysis and drafted the manuscript. KN. H. segregated T cell subpopulations, performed the validation experiments in clinical samples and in vitro, performed some statistical analyses, and reviewed the manuscript. N. W. prepared the single‐cell transcriptome, performed the animal experiments, and reviewed the manuscript; SS. L. and XG. C. collected information about the subjects, performed some statistical analyses, and reviewed the manuscript; X. Y., XJ. C., T. T., and R. G. provided assistance for cell isolation, culture, and treatment and reviewed the manuscript; XY. L. designed the study, provided the funding, and supervised the study. All authors have read and approved the final manuscript.

## Ethics Statement

This clinical study was approved by the Ethics Committee of Xi'an JiaoTong University (XJTU‐2022018). All patients and controls involved in this study provided informed consent. This animal experiments were approved by the Ethics Committee of Xi'an JiaoTong University (XJTULAC‐2022018).

## Conflicts of Interest

The authors declare no conflicts of interest.

## Supporting information




**Supporting File**: mco270311‐sup‐0001‐SuppMat.pdf

## Data Availability

The m5C‐seq and scRNA‐seq are available from the online publicly accessible database CNCB‐NGDC (#0006305). All experimental data, R, and other custom scripts are available from the corresponding author upon request.
