# Supplementary material for: Nanoparticle‐Delivered siRNA Targeting NSUN4 Relieves Systemic Lupus Erythematosus through Declining Mitophagy‐Mediated CD8+T Cell Exhaustion
Source: MedComm (2020). 2025 Aug 3;6(8):e70311. doi: 10.1002/mco2.70311 (PMC12318824; doi:10.1002/mco2.70311)
Supplement: Supplementary file 1 — Supporting File: mco270311‐sup‐0001‐SuppMat.pdf [file MCO2-6-e70311-s001.pdf]

Nanoparticle-delivered siRNA targeting NSUN4 relieves systemic lupus erythematosus through declining mitophagy-mediated CD8+T cell exhaustion

Bincheng Ren [1,#], Kaini He [2,#], Ning Wei [3,#], Shanshan Liu [1], Xiaoguang Cui [1], Xin Yang [1], Xiaojing Cheng [1], Tian Tian [1], Ru Gu [4], Xueyi Li [1,*]

[1] Department of Rheumatology and Immunology, the Second Affiliated Hospital of Xi'an Jiaotong University, Xi'an 710004, China

[2] Department of Gastroenterology, the Second Affiliated Hospital of Xi'an Jiaotong University, Xi'an 710004, China

[3] College of Animal Science and Technology, Northwest A&F University, Yangling 712100, China

[4] Department of Anesthesiology, the Second Affiliated Hospital of Xi'an Jiaotong University, Xi'an 710004, China

[#]*These authors contributed equally to this study.*

**Correspondence to:*

Prof. Xueyi Li, Department of Rheumatology and Immunology, the Second Affiliated Hospital of Xi'an Jiaotong University, 157 Xiwu Road, Xi'an 710004, China; Tel: 86-13992891987; Fax: 86-29-87679311; Email: lixueyi@mail.xjtu.edu.cn.

Supplementary figures

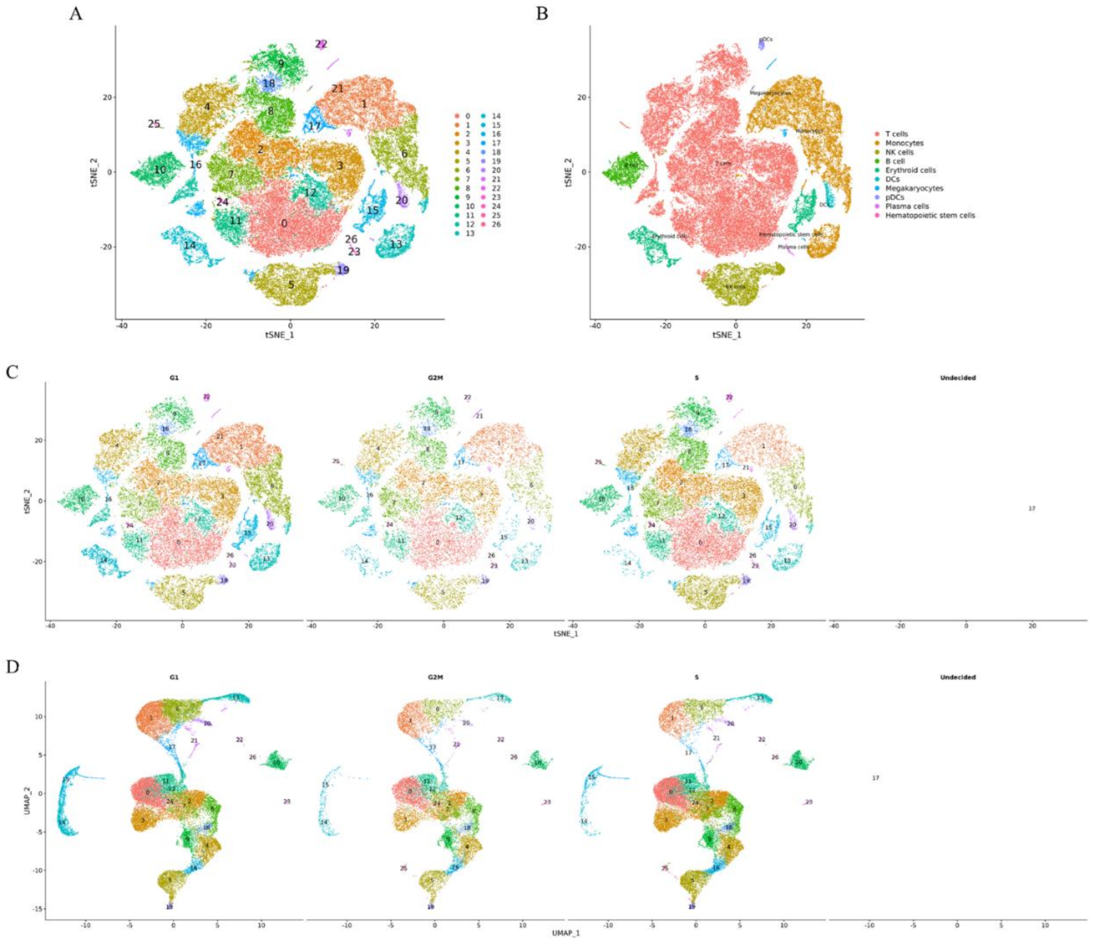

Figure S1. UMAP nonlinear clustering and t-SNE dimensionality reduction clustering assays for the blood cells of overall samples. A. t-SNE dimensionality reduction clustering assay for cell clusters in the blood cells of overall samples. B. t-SNE dimensionality reduction assay for cell types in the blood cells of overall samples. C. t-SNE dimensionality reduction assay for cell clusters at different cell cycle stages in the blood cells of overall samples. D. UMAP nonlinear clustering assay for cell clusters at different cell cycle stages in the blood cells of overall samples.

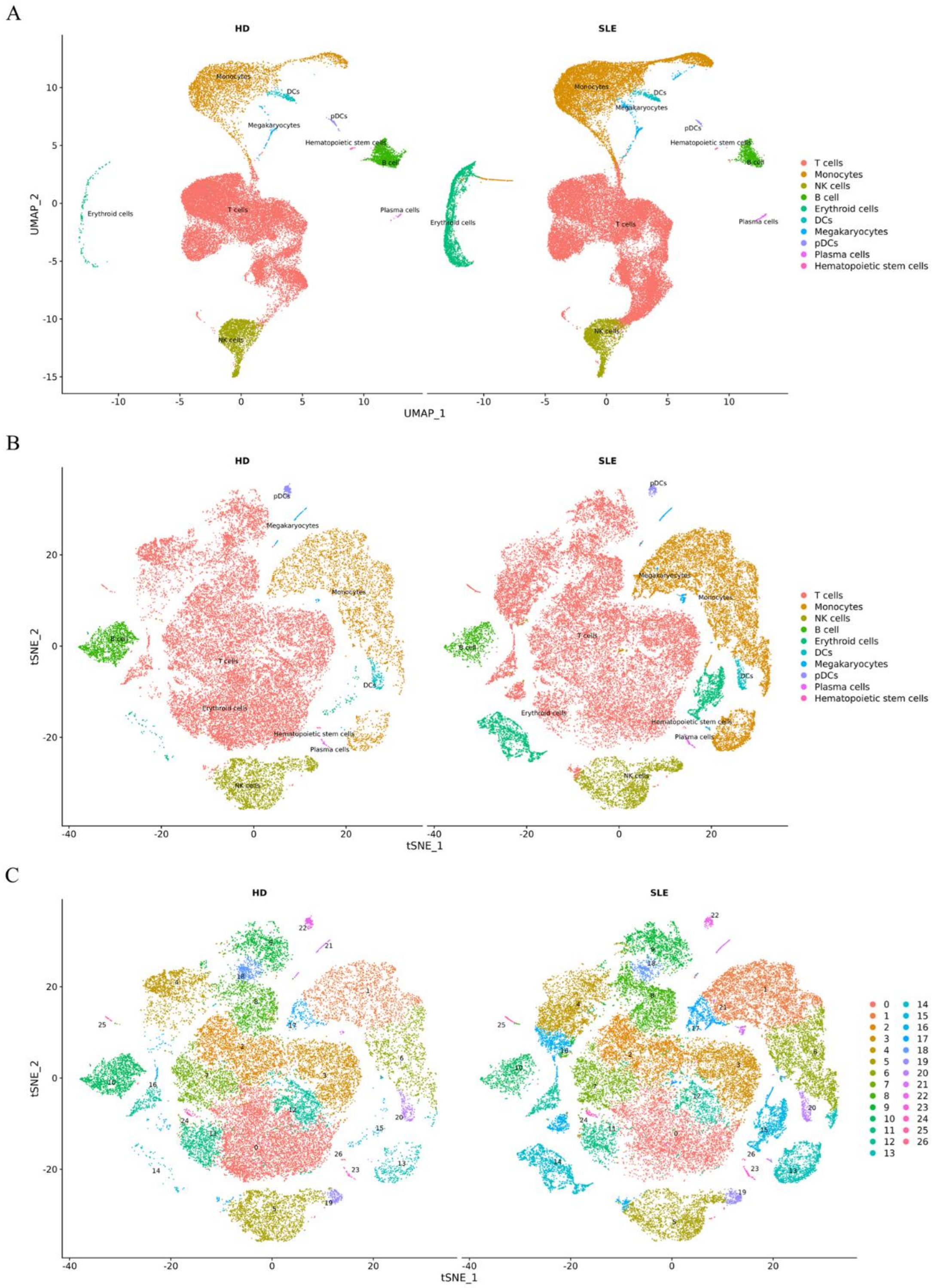

Figure S2. UMAP nonlinear clustering and t-SNE dimensionality reduction clustering assays for the blood cells between SLE patients and healthy controls. A. UMAP nonlinear clustering assay for cell types in the blood cells of between SLE patients and healthy controls. B. t-SNE dimensionality reduction assay for cell types in

the blood cells between SLE patients and healthy controls. C. t-SNE dimensionality reduction assay for cell clusters in the blood cells between SLE patients and healthy controls.

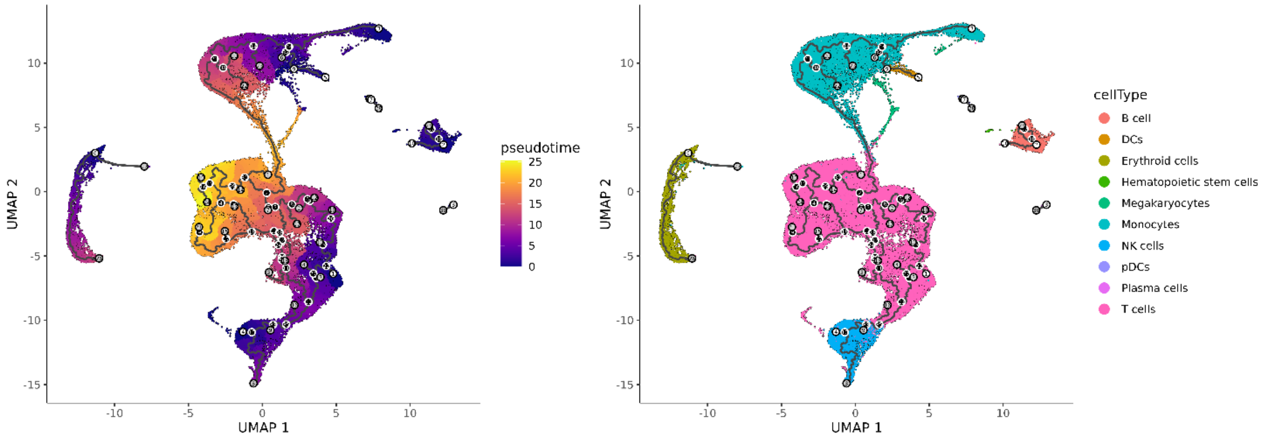

Figure S3. Quasi temporal (Pseudotime) analysis was performed using R package Monocle3 based on UMAP dimensionality reduction in overall blood cells (left) or each cell type (right).

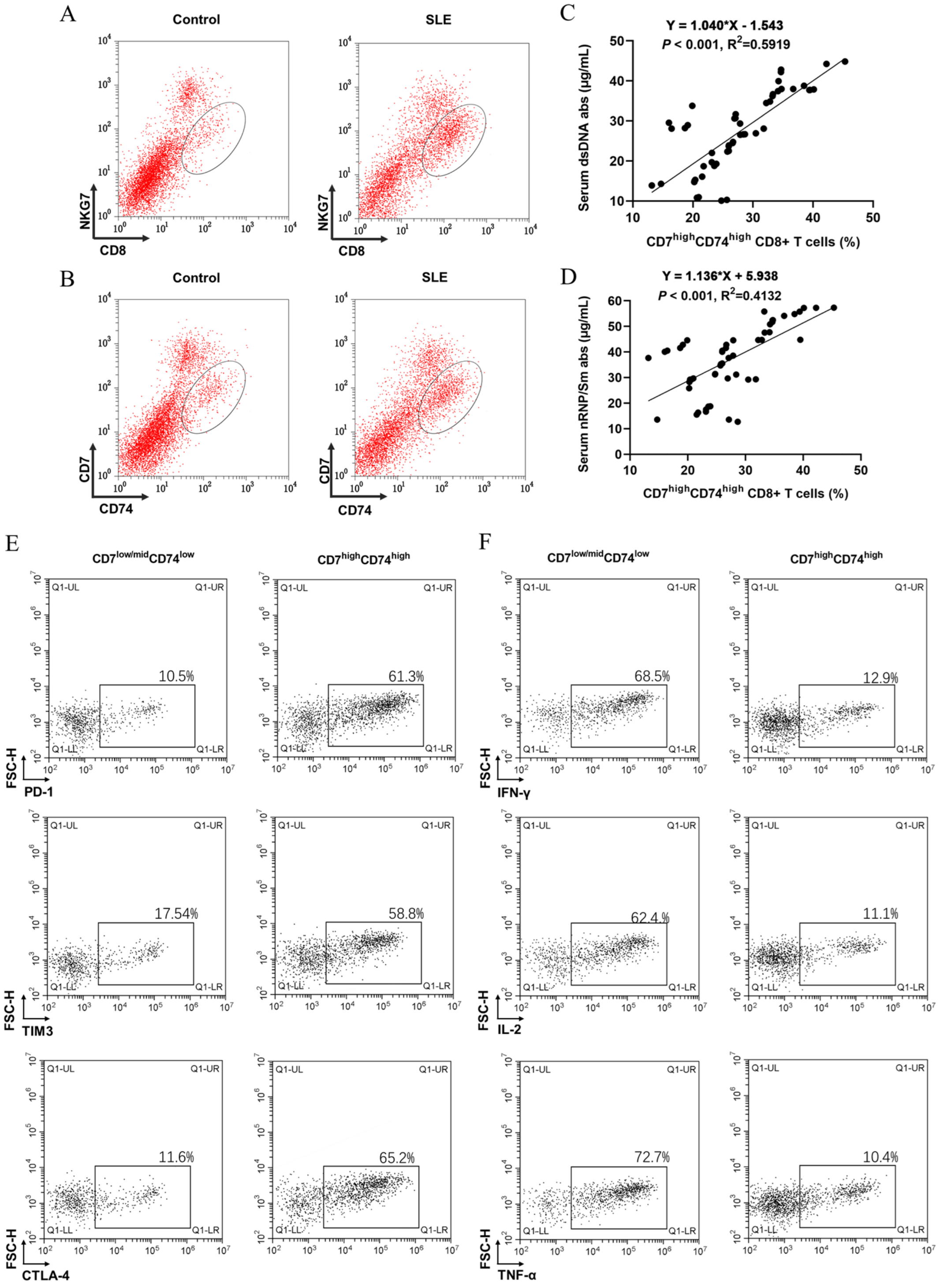

Figure S4. The correlation of CD7^{high}CD74^{high} CD8+T cell proportion with SLE diagnostic indicators, and their immune endocrine characteristics. Fluorescence activated cell sorting (FACS) was used to assort (A) NKG7^{+}CD8^{+} T cells from total T cells and (B) CD7^{high}CD74^{high} from NKG7^{+}CD8^{+} T cells. Pearson correlation coefficient was used to analyze the associations of CD7^{high}CD74^{high} CD8+T cell

proportion with the levels of serum anti-double DNA antibodies (C, anti-dsDNA abs) and anti-Nuclear ribonuclear protein/Smith antibodies (D, anti-nRNP/Sm abs). E. The percentages of IFN-γ^{high}, IL-2^{high} and TNF-α^{high} cells in $CD7^{high}CD74^{high}$.

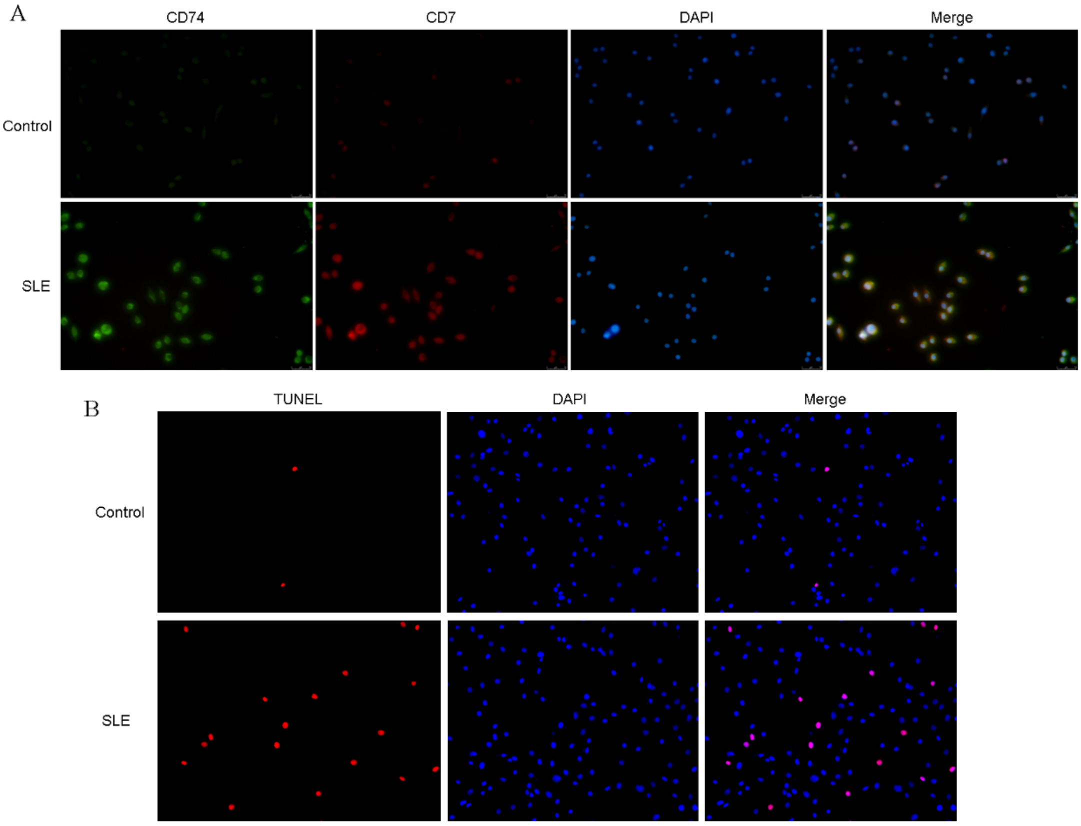

Figure S5. The exhausted CD8+ T cells in SLE patients displayed high levels of CD7 and CD74 and cell apoptosis. A. Dual immunofluorescence staining was used to evaluate the CD7 and CD74 levels in CD8+NKG7+ T cells from Controls and SLEs. B. TUNEL staining revealed that the apoptosis level of CD8+NKG7+ T cells from Controls and SLEs. DAPI was used to label the nucleus (blue). × 200.

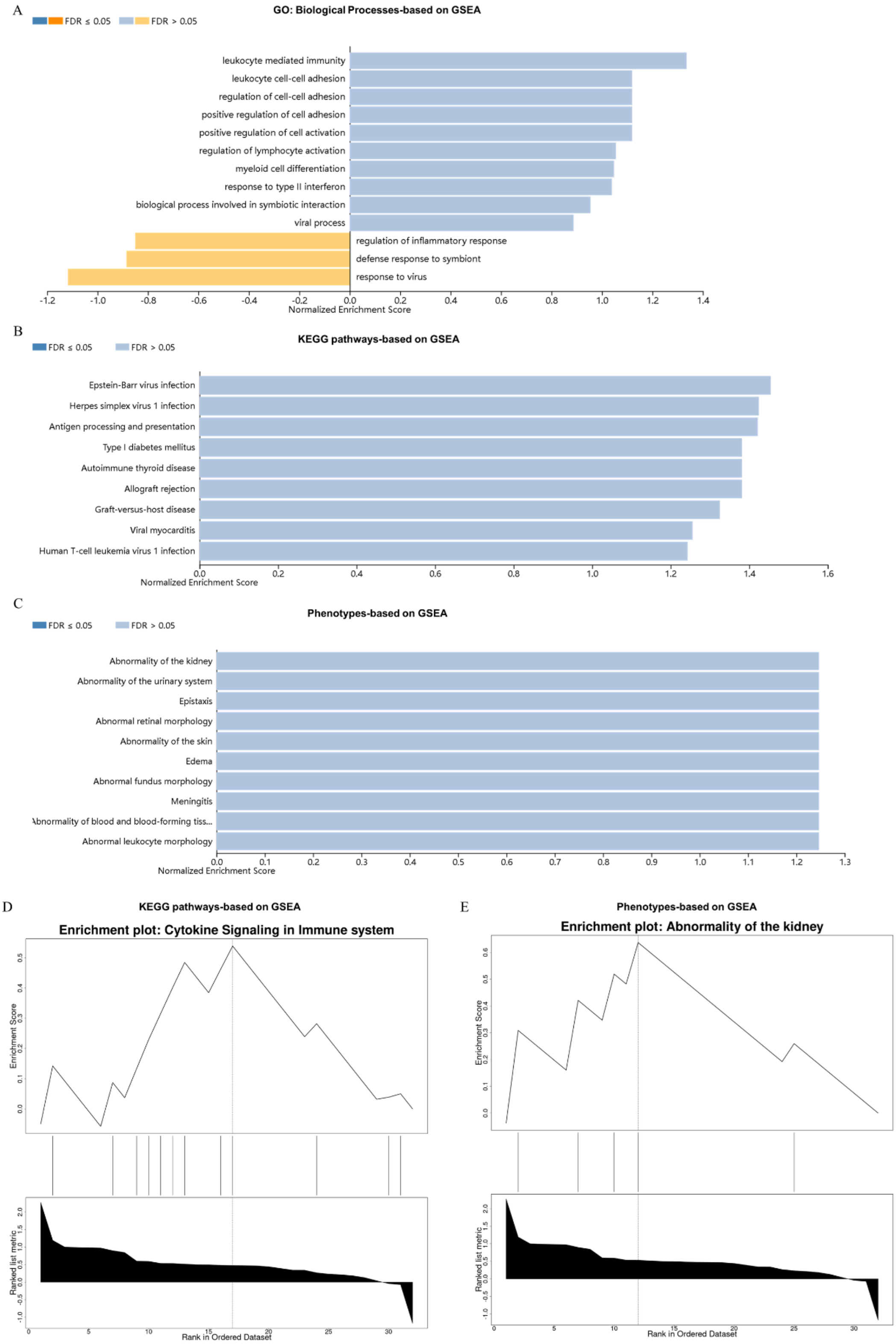

Figure S6. Gene Set Enrichment Analysis (GSEA) was used to annote the functions of the DEGs of cluster 16. The sequencing information of DEGs from cluster 16 were analyzed by the online website WEB-based GEne SeT AnaLysis Toolkit for (A)

GO>biological processes, (B) KEGG pathways, and (C) Top 10 phenotypes, with the method Gene Set Enrichment Analysis. The details of the most associted (D) pathway and (E) phenotype were output.

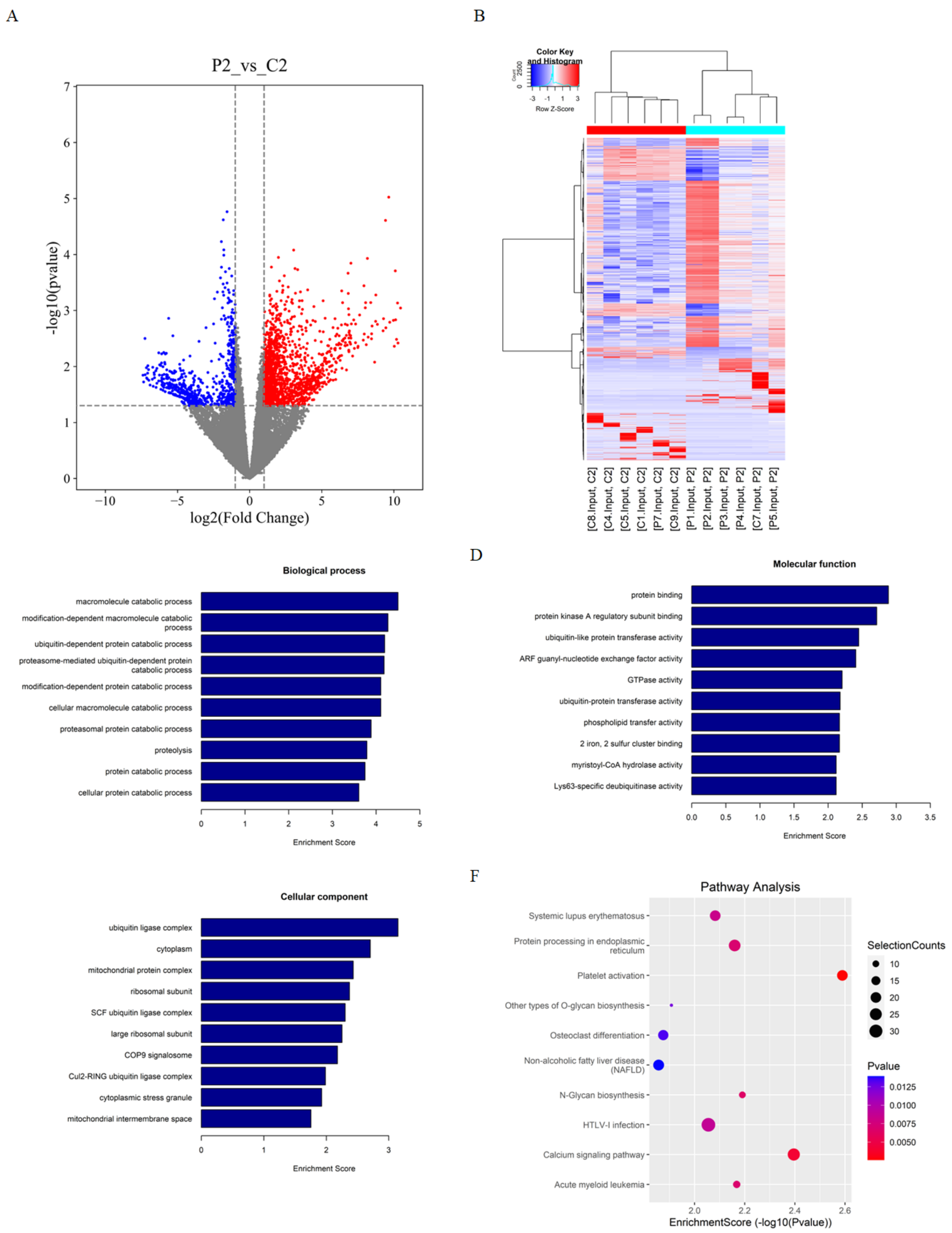

Figure S7. RNA-seq analysis for the DEGs between C_2 and P_2 and their functional annotation. RNA high-throughput sequencing service was CBCs from 6 SLE patients

(P_2) and 6 healthy controls (C_2). EdgeR was used for standardization and calculation of the multiple changes and p-value between the two groups of samples to screen differentially expressed genes (DEGs). A and B. Volcano plots and Heatmap of the DEGs. C-E. GO analysis for the biological processes, molecular functions and cellular components of the DEGs. F. KEGG analysis for the related signaling pathways of the DEGs.

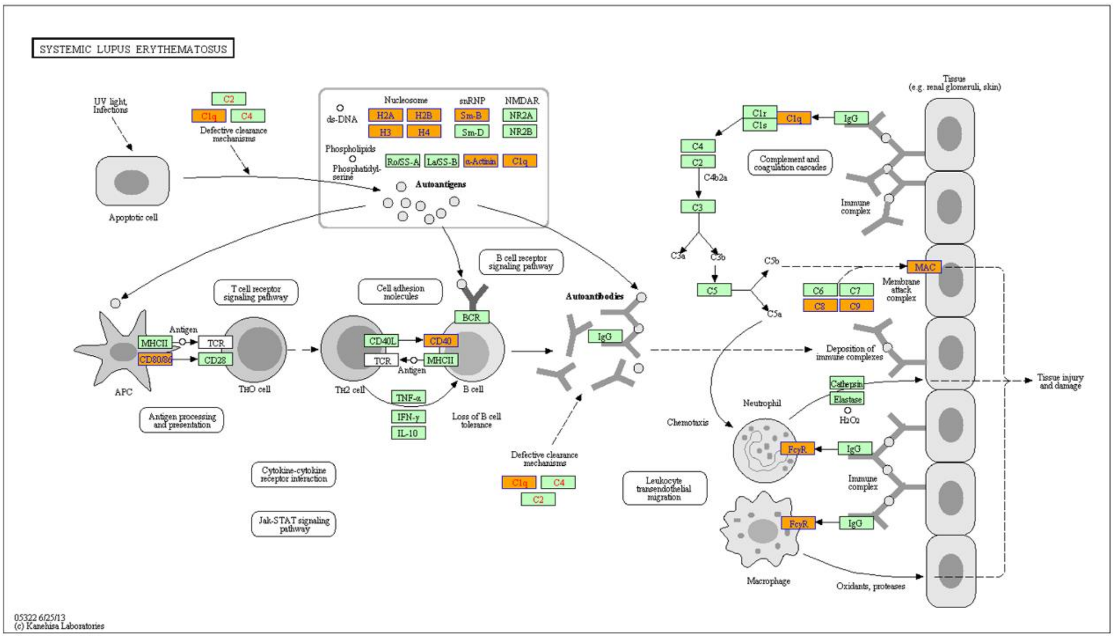

Figure S8. Details of SLE pathway figured out from KEGG analysis.

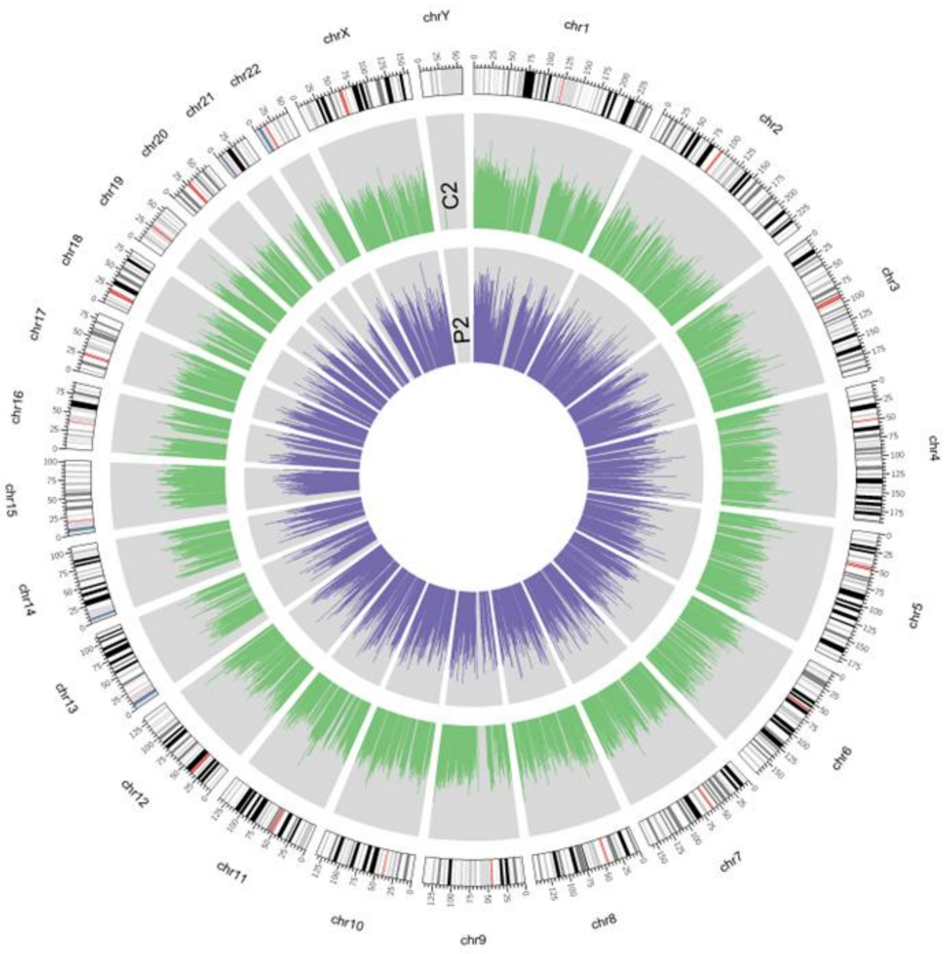

Figure S9. Detailed distribution of DEGs and DMGs in each chromosome.

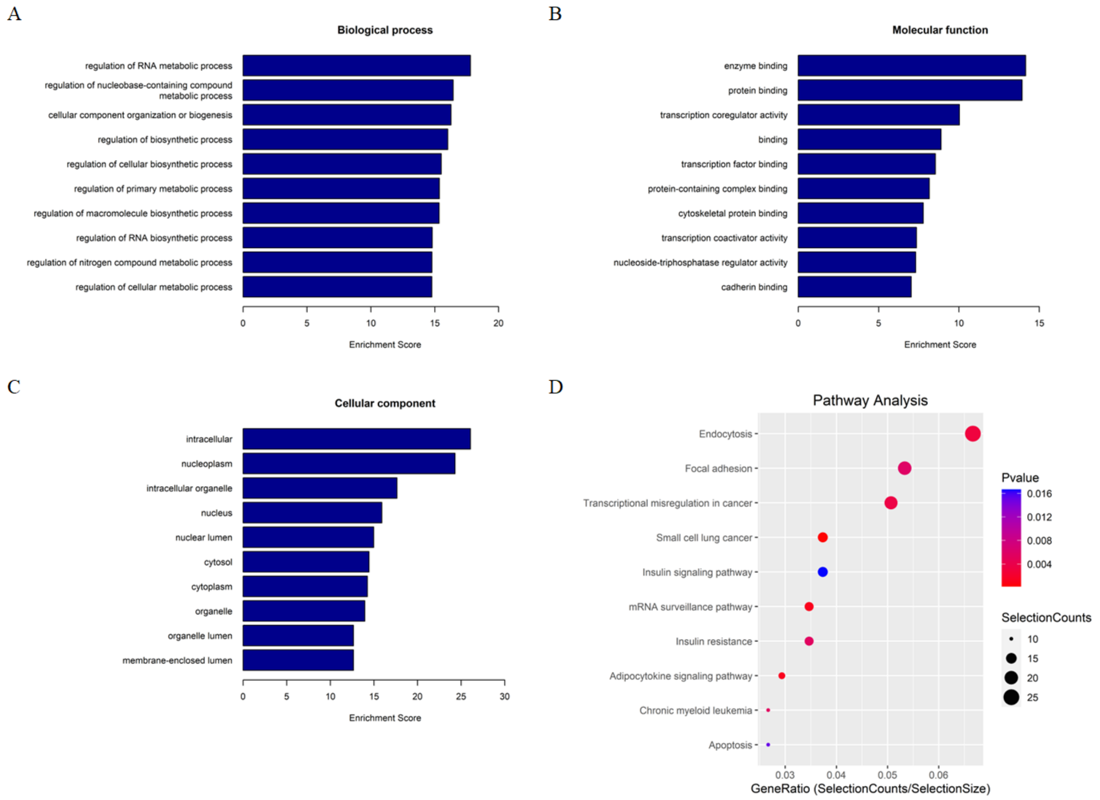

Figure S10. GO and KEGG analyses for functions of the DMGs. A-C. GO analysis for the biological processes, molecular functions and cellular components of the DMGs. D. KEGG analysis for the related signaling pathways of the DMGs.

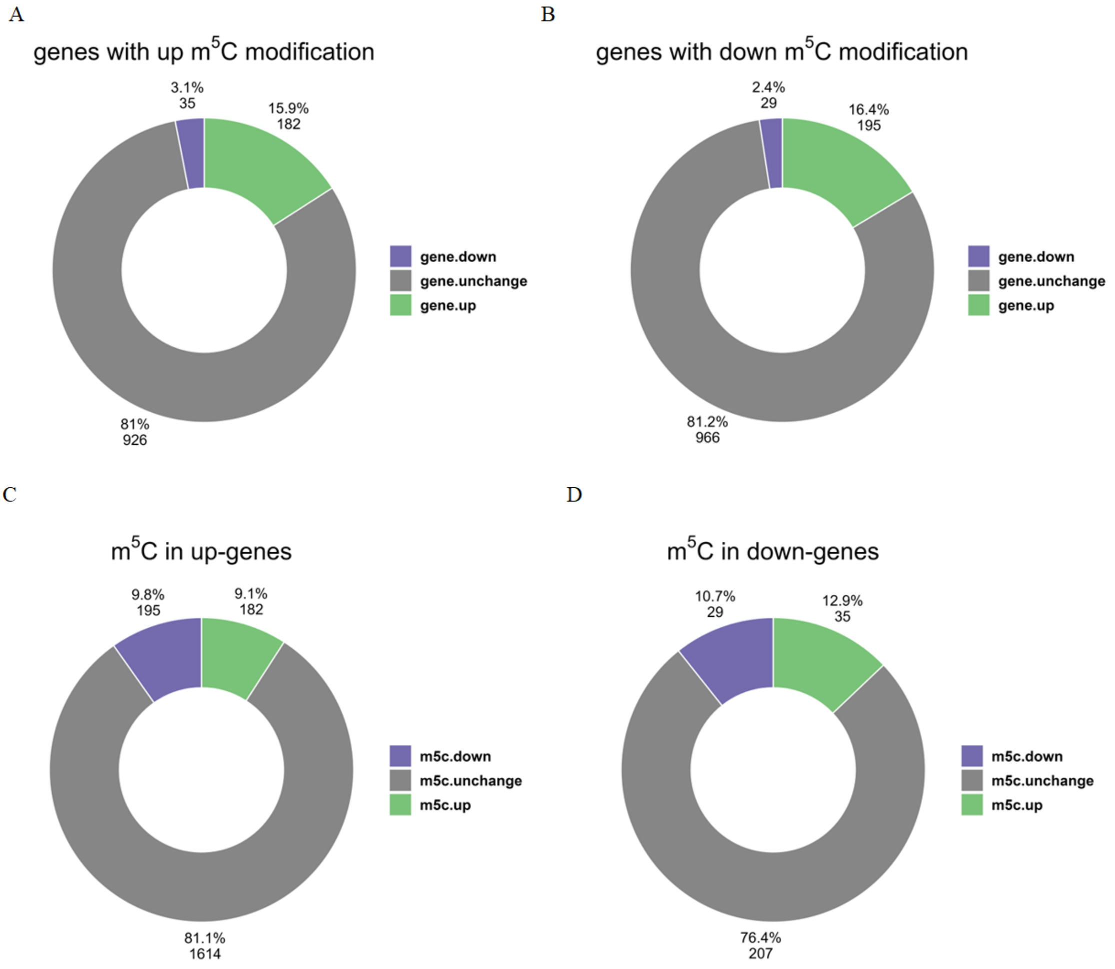

Figure S11. Intersection analysis of the DEGs and DMGs. A. The percentages of downregulated, unchanged and upregulated genes in hypermethylated genes. D. The percentages of downregulated, unchanged and upregulated genes in hypomethylated genes. C. The percentages of hypermethylated, unchanged and hypomethylated genes in upregulated genes. D. The percentages of hypermethylated, unchanged and hypomethylated genes in downregulated genes.

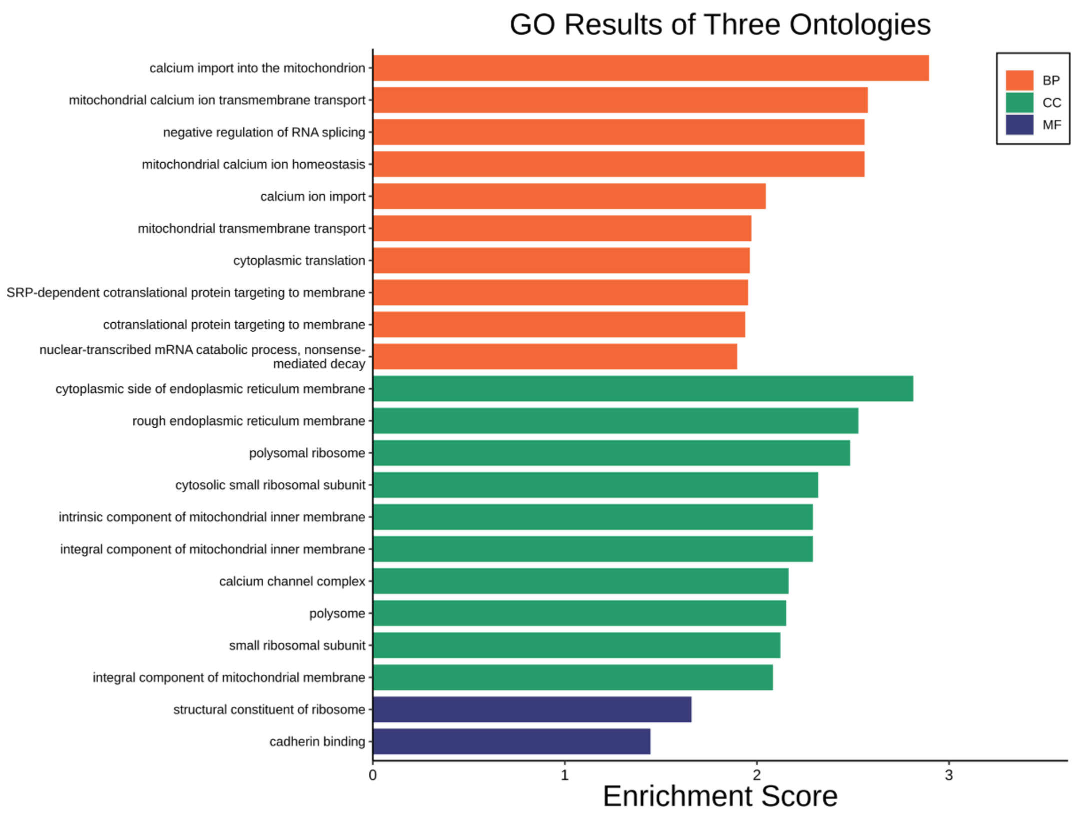

Figure S12. GO analysis for the biological processes, molecular functions and cellular components of the top 15 DEGs in cluster 16 T cells.

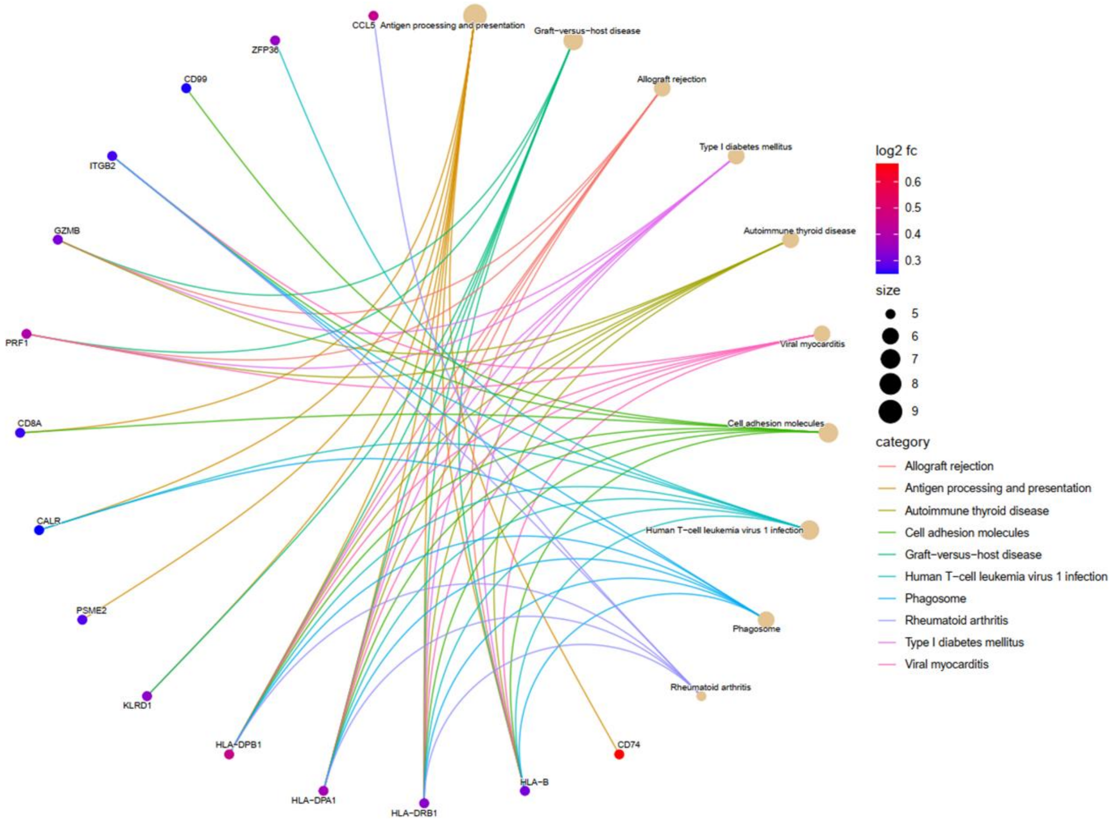

Figure S13. KEGG analysis for the related pathways of the top 15 DEGs in cluster 16 T cells.

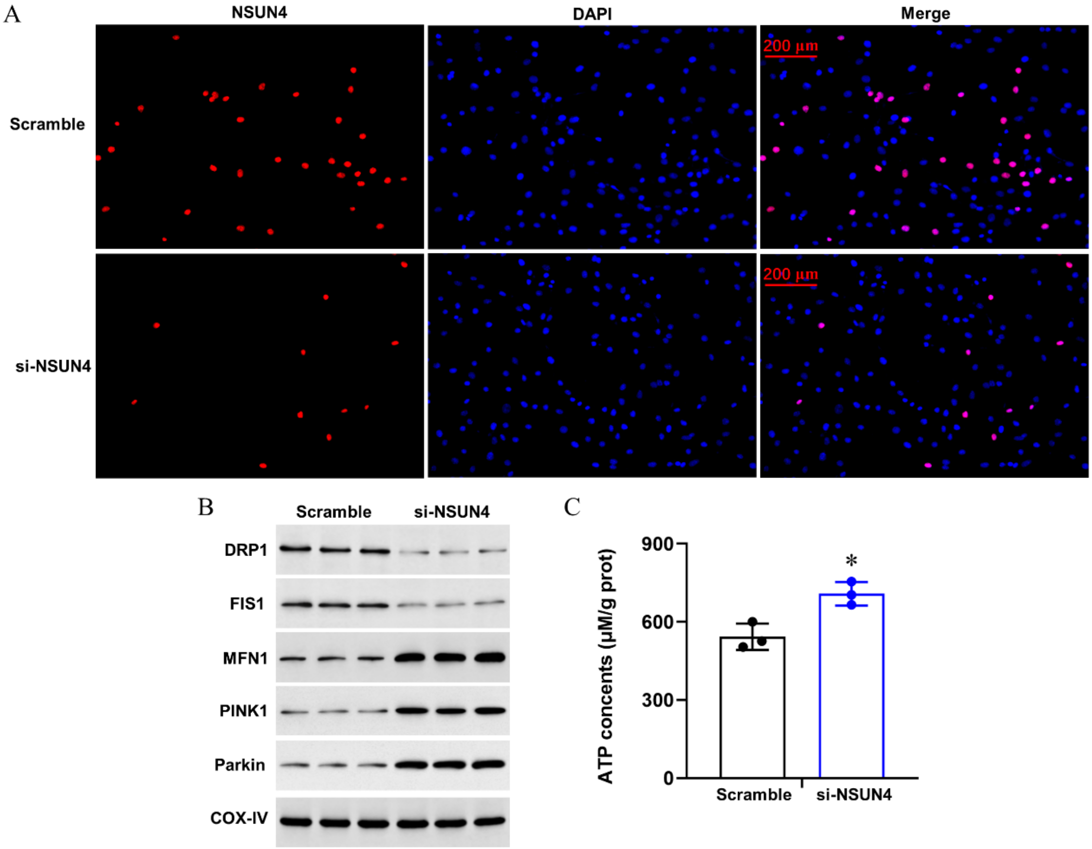

Figure S14. Knockdown of NSUN4 suppressed cell apoptosis and mitochondrial dysfunction in cultured CD8+T cells. NSUN4 was knocked down in cultured CD8+T cells by transfection of 50 nM NSUN4 siRNA. A. TUNEL staining was used to evaluate cell apoptosis. DAPI was used to mark the nucleus (blue). × 200. B. Western blot was used to detect the protein levels of mitochondrial markers and mitophagy markers, including DRP1, FIS1, MFN1, PINK1 and Parkin. C. ATP production was detected with an ATP Chemiluminescence Assay Kit. N = 3, **P* < 0.05, ***P* < 0.01 compared with Scramble.

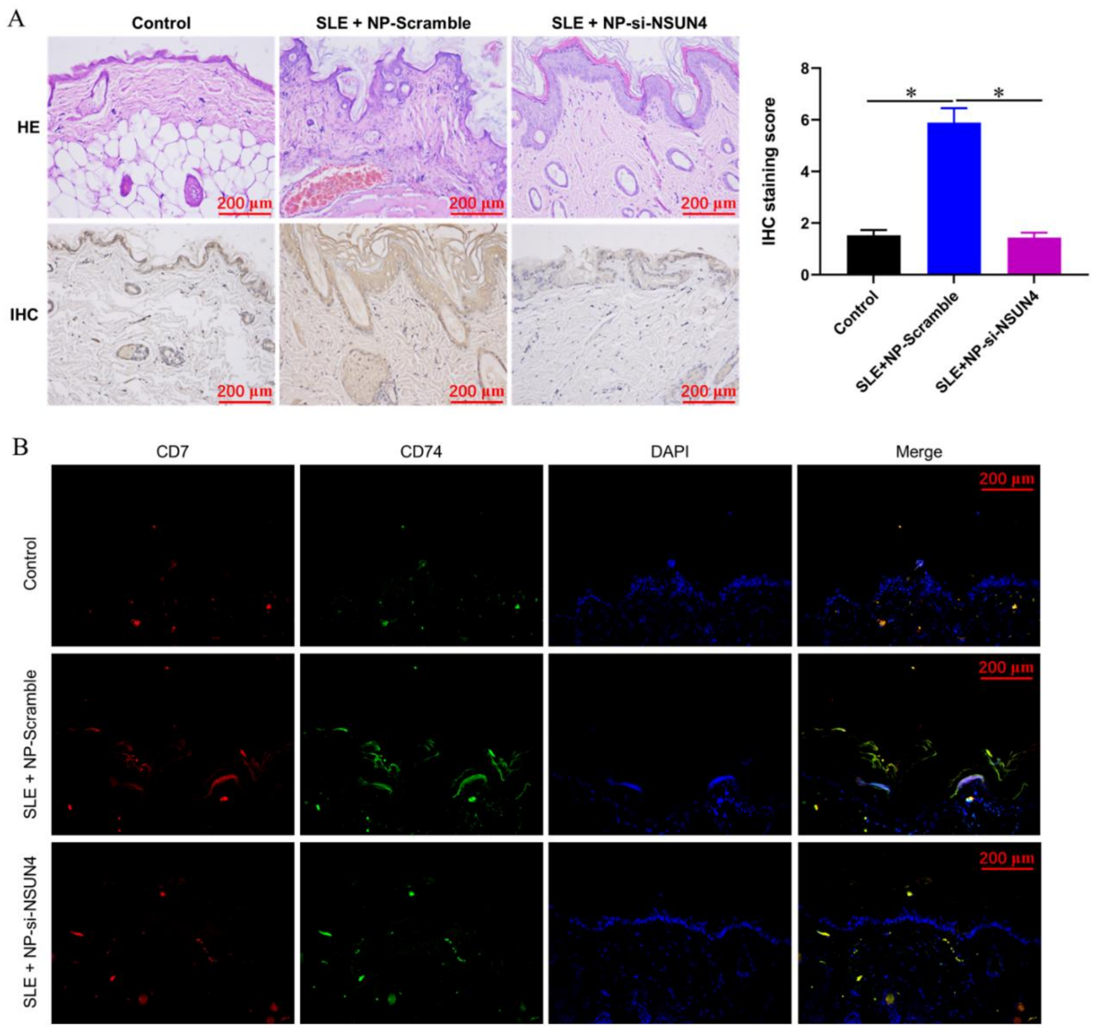

Figure S15. The effect of NSUN4 downregulation on the histopathology and infiltration of $CD7^{high}CD74^{high}$ CD8+T cells in the skin in spontaneous SLE mice. Spontaneous lupus MRL/lpr mice applied to investigate the role of NSUN4 in SLE progression. Nanoparticle-delivered siRNA against NSUN4 was administrated into MRL/lpr mice by the tail vein injection and the Scramble was used as a negative control. After blood collection, the skin tissues were spliced, and hematoxylin-eosin (HE), immunohistochemistry (IHC) and dual immunofluorescence were used to observe the (A) histopathology and NSUN4 expression level, and (B) infiltration of $CD7^{high}CD74^{high}$ T cells in the skin. N = 6.

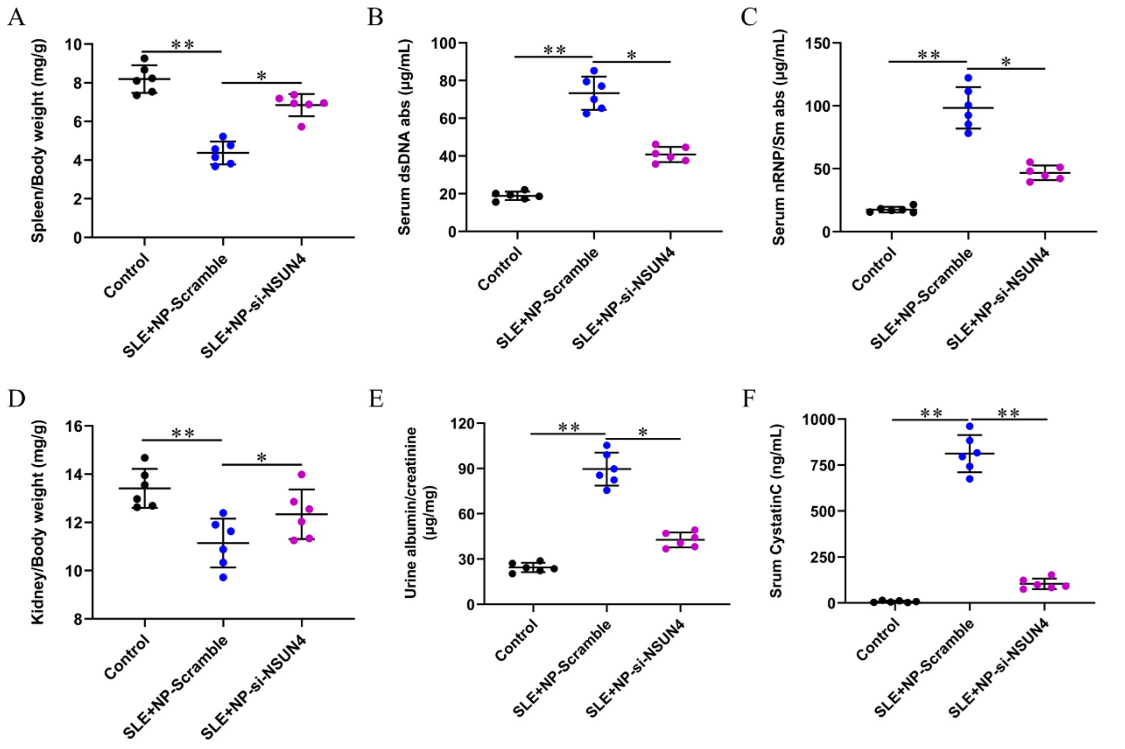

Figure S16. The effect of NSUN4 downregulation on serum autoimmune antibody levels and kidney function in SLE mice. Spontaneous lupus MRL/lpr mice applied to investigate the role of NSUN4 in SLE progression. Nanoparticle-delivered siRNA against NSUN4 was administrated into MRL/lpr mice by the tail vein injection and the Scramble was used as a negative control. Kidney function and contents of serum autoimmune antibodies were evaluated by measuring the indexes as follows: A. Spleen weight index; B and C. Serum dsDNA antibodies and nRNP/Sm antibodies; D. Kidney weight index; E. Urine albumin/creatinine ratio; F. Serum cystatin C content. N = 6, *$P < 0.05$, **$P < 0.01$.

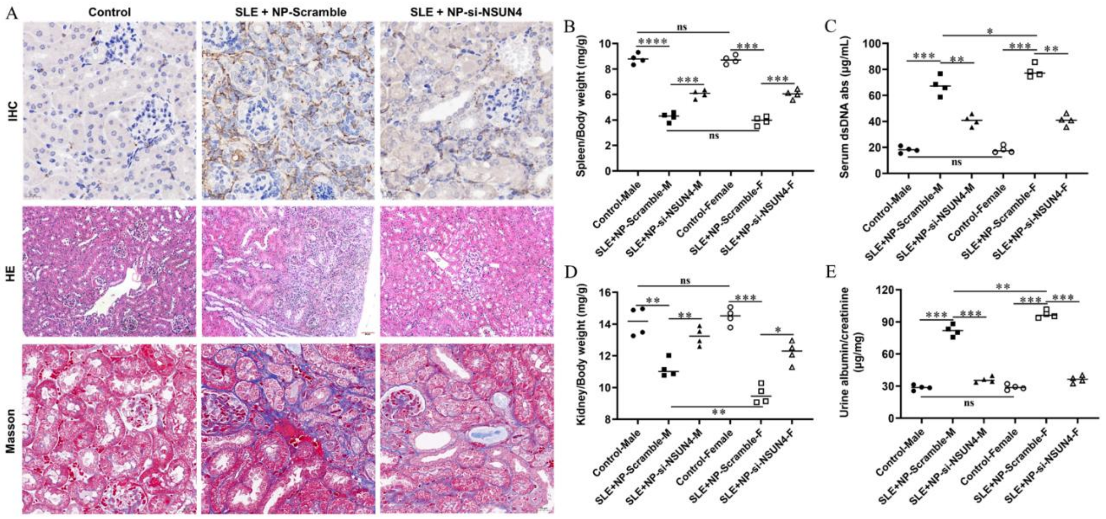

Figure S17. Knockdown of NSUN4 alleviates kidney damage in pristane-induced SLE mice. Induced SLE model was established with the intraperitoneal injection of 0.5 mL of pristane in SJL/J mice verify the role of NSUN4 in SLE progression. Liposome-protamine-hyaluronic acid (LPH) nanoparticle (NP)-delivered siRNA against NSUN4 was administrated into pristane-induced SLE mice by the tail vein injection and the NP-Scramble was used as a negative control. A. The kidney tissues were collected, spliced and stained with HE, Masson, and immunohistochemistry to observe the tissue morphology and NSUN4 expression. Kidney function and contents of serum autoimmune antibodies were evaluated by measuring: B. Spleen weight index; C. Serum dsDNA antibodies and nRNP/Sm antibodies; D. Kidney weight index; E. Urine albumin/creatinine ratio; The proportions of NKG7+CD8+ T cells (left) and CD7^{high}CD74^{high} CD8+T cells (right) in each group. N = 4, **P* < 0.05, ***P* < 0.01, ****P* < 0.001, *****P* < 0.0001; ns represents *P* > 0.05.

Supplementary Tables

Table S1. Statistics of the number of cells in each sample in single-cell sequencing.

seq_folder	0	1	2	3	4	5	6	7	8	9	10	11	12	13	14	15	16	17	18	19	20	21	22	23	24	25
aHD4-F	922	336	828	363	517	776	176	435	470	393	626	216	140	92	5	1	38	50	89	47	39	20	18	15	10	9
aHD5-F	937	1068	619	715	322	765	740	439	353	269	527	198	255	218	58	52	65	141	100	69	67	33	116	10	12	15
aHD6-F	4152	250	1027	957	810	544	156	725	366	363	482	943	737	70	7	15	60	60	207	72	74	38	34	22	47	9
aHD7-F	2674	509	710	678	550	695	357	533	396	342	545	452	377	127	23	23	54	84	132	63	60	30	56	16	23	11
aHD8-F	2130	192	538	960	260	571	248	480	446	33	538	294	458	151	22	29	17	107	17	82	26	3	88	5	3	10
aHD9-F	1654	116	310	350	661	924	427	575	608	16	351	109	174	87	12	5	50	86	76	18	85	41	20	3	11	16
aHD10-F	1525	99	199	35	269	49	145	187	586	366	616	503	88	196	16	19	33	114	94	91	90	24	80	21	36	13
aHD11-M	3466	497	655	948	729	633	191	251	603	13	552	286	54	141	19	4	31	127	90	74	87	14	60	1	16	1
aHD12-M	1578	361	597	873	236	460	541	274	461	573	727	191	566	50	22	11	44	114	31	50	100	14	86	10	15	18
aHD13-M	2041	504	178	57	400	70	233	168	392	275	139	176	142	168	9	5	4	130	31	1	93	12	17	7	14	3
aHD14-M	2421	126	796	841	252	159	117	449	474	16	810	298	186	115	7	22	83	44	24	52	20	23	13	21	12	6
Mean	2136.4	368.9	587.0	616.1	455.1	513.3	302.8	410.5	468.6	241.7	537.5	333.3	288.8	128.6	18.2	16.9	43.5	96.1	81.0	56.3	67.4	22.9	53.5	11.9	18.1	10.1
Mean-F	1999.1	367.1	604.4	579.7	484.1	617.7	321.3	482.0	460.7	254.6	526.4	387.9	318.4	134.4	20.4	20.6	45.3	91.7	102.1	63.1	63.0	27.0	58.9	13.1	20.3	11.9
Mean-M	2376.5	372.0	556.5	679.8	404.3	330.5	270.5	285.5	482.5	219.3	557.0	237.8	237.0	118.5	14.3	10.5	40.5	103.8	44.0	44.3	75.0	15.8	44.0	9.8	14.3	7.0
aSLE1-F	245	354	183	283	724	376	284	108	173	335	63	58	75	201	91	127	377	30	28	115	17	47	16	16	12	53
aSLE2-F	189	760	211	167	213	131	681	153	241	190	55	54	84	500	256	261	199	141	67	22	36	76	10	30	8	24
aSLE3-F	1205	775	604	793	287	137	471	496	500	309	283	302	351	398	21	6	64	155	57	30	69	36	22	45	15	11
aSLE4-F	748	778	528	486	570	573	483	281	278	236	96	197	173	164	4	7	71	157	78	45	62	89	39	35	4	13
aSLE5-F	610	340	282	584	447	419	299	168	209	142	146	141	145	91	1	1	339	64	34	25	23	13	8	6	27	18
aSLE6-F	442	608	494	265	178	58	363	276	337	150	21	115	99	106	19	31	32	65	3		33	15	13	6	34	4
aSLE7-F	341	530	309	113	214	352	268	203	254	178	130	125	114	142	1180	1010	210	236	35	31	34	53	12	1	9	0
aSLE8-F	57	515	257	184	510	19	60	325	212	210	38	13	45	265	354	107	282	53	71	57	40	58	20	33	16	8
aSLE9-F	185	245	222	243	334	65	499	336	339	75	140	218	85	56	35	258	192	155	23	70	37	73	13	32	6	22
aSLE10-M	300	630	482	354	496	329	495	381	448	212	147	214	183	272	85	189	276	28	21	26	25	61	21	27	16	13
aSLE11-M	158	465	277	103	178	170	122	210	154	64	12	122	162	95	283	218	260	0	15	37	43	23	1	19	2	23
aSLE12-M	333	354	336	121	473	57	461	310	21	306	44	60	163	39	281	334	247	199	53	27	63	35	26	8	11	26
aSLE13-M	417	321	265	350	393	374	647	201	258	183	45	148	123	7	26	186	49	161	4	20	15	67	3	10	18	21
Mean	402.3	513.5	342.3	311.2	385.9	235.4	394.8	265.2	263.4	199.2	93.8	135.9	138.6	179.7	202.8	210.4	199.8	111.1	37.6	42.1	38.2	49.7	15.7	20.6	13.7	18.2
Mean-F	446.9	545.0	343.3	346.4	386.3	236.7	378.7	260.7	282.6	202.8	108.0	135.9	130.1	213.7	217.9	200.9	196.2	117.3	44.0	49.4	39.0	51.1	17.0	22.7	14.6	17.0
Mean-M	302.0	442.5	340.0	232.0	385.0	232.5	431.3	275.5	220.3	191.3	62.0	136.0	157.8	103.3	168.8	231.8	208.0	97.0	23.3	27.5	36.5	46.5	12.8	16.0	11.8	20.8
Sum-ALL	28730	10733	10907	10823	10023	8706	8464	7964	8579	5249	7133	5433	4979	3751	2836	2921	3077	2501	1380	1124	1238	898	792	399	377	347
Mean-ALL	1197.1	447.2	454.5	451.0	417.6	362.8	352.7	331.8	357.5	218.7	297.2	226.4	207.5	156.3	118.2	121.7	128.2	104.2	57.5	46.8	51.6	37.4	33.0	16.6	15.7	14.5

Table S2. DEGs in cluster 16.

P_val	avg_log2FC	pct.1	pct.2	P_val_adj	gene	up_down
0	0.44398288	0.452	0.2	0	ISG15	upregulated
0	0.28357913	0.909	0.749	0	SH3BGRL3	upregulated
0	0.4266104	0.373	0.133	0	IFI6	upregulated
0	0.51721144	0.295	0.033	0	IFI44L	upregulated
0	0.3816722	0.556	0.32	0	ID2	upregulated
0	0.32728194	0.18	0.048	0	GNLY	upregulated
0	0.25535528	0.746	0.533	0	NBEAL1	upregulated
0	0.41835087	0.162	0.013	0	FGFBP2	upregulated
0	0.30016163	0.198	0.053	0	HOPX	upregulated
0	0.51438146	0.308	0.115	0	GZMA	upregulated
0	0.66789513	0.635	0.2	0	**CD74**	upregulated
0	0.30958865	0.998	0.978	0	HLA-B	upregulated
0	0.29303979	0.533	0.335	0	CLIC1	upregulated
0	0.33576847	0.154	0.007	0	HLA-DRB1	upregulated
0	0.29690613	0.516	0.302	0	PSMB9	upregulated
0	0.377448	0.184	0.006	0	HLA-DPA1	upregulated
0	0.45528261	0.285	0.053	0	HLA-DPB1	upregulated
0	0.44733786	0.703	0.424	0	LY6E	upregulated
0	0.3297282	0.782	0.556	0	SRGN	upregulated
0	0.39753005	0.196	0.02	0	PRF1	upregulated
0	0.42920551	0.766	0.478	0	IFITM2	upregulated
0	0.54101144	0.61	0.253	0	IFITM1	upregulated
0	0.28301141	0.897	0.714	0	GAPDH	upregulated
0	0.34010657	0.154	0.01	0	KLRD1	upregulated
0	-0.35941091	0.92	0.99	0	RPS26	downregulated
0	0.38829033	0.261	0.043	0	EPSTI1	upregulated
0	0.6256806	0.23	0.028	0	DUSP2	upregulated
0	0.31117055	0.128	0.007	0	RGS1	upregulated
0	0.26258932	0.301	0.131	0	PTGER2	upregulated
0	0.36756554	0.127	0.003	0	IFI27	upregulated
0	0.27096451	0.294	0.127	0	ANXA2	upregulated
0	0.38537242	0.48	0.243	0	MT2A	upregulated
0	0.27042238	0.233	0.074	0	XAF1	upregulated
0	0.43060946	0.595	0.286	0	EIF5A	upregulated
0	0.46555388	0.201	0.023	0	CCL4	upregulated
0	0.50650607	0.296	0.092	0	CST7	upregulated
0	0.31518258	0.344	0.15	0	MYO1F	upregulated
0	0.35010053	0.786	0.563	0	ZFP36	upregulated
0	0.86452179	0.336	0.053	0	**CD7**	upregulated

0	0.32289085	0.333	0.165	0	LGALS1	upregulated
0	-0.28918294	0.45	0.585	0	SMDT1	downregulated
0	0.25767466	0.21	0.063	0	MX1	upregulated
2.693E-305	0.26813716	0.275	0.123	5.077E-301	CTSC	upregulated
1.548E-303	0.26226795	0.366	0.187	2.919E-299	BST2	upregulated
1.072E-300	0.27685016	0.544	0.339	2.02E-296	PSME2	upregulated
4.808E-299	0.25592921	0.305	0.144	9.063E-295	FLNA	upregulated
4.53E-287	0.25903423	0.674	0.469	8.539E-283	PPP1R15A	upregulated
2.616E-279	0.26067847	0.57	0.36	4.932E-275	RHOA	upregulated
1.569E-277	0.26905124	0.618	0.421	2.958E-273	ITGB2	upregulated
5.175E-276	0.44497375	0.379	0.249	9.756E-272	CCL5	upregulated
1.245E-275	0.26532405	0.424	0.244	2.347E-271	S100A11	upregulated
1.461E-270	0.25182035	0.244	0.106	2.755E-266	AC092580.4	upregulated
9.769E-270	0.26296547	0.301	0.152	1.842E-265	C12orf75	upregulated
1.165E-260	0.25844823	0.66	0.476	2.197E-256	IER2	upregulated
5.369E-251	0.25072975	0.482	0.294	1.012E-246	CALR	upregulated
2.356E-242	0.25393611	0.553	0.37	4.442E-238	CD99	upregulated
1.619E-241	0.26251607	0.301	0.158	3.052E-237	CD8A	upregulated
8.752E-238	0.26428767	0.567	0.389	1.65E-233	GZMB	upregulated
1.747E-213	0.26558859	0.354	0.21	3.294E-209	GZMH	upregulated

Table S3. Primers used in this study.

For qRT-PCR	Primer sequence (5' → 3')
NSUN4-Forward Primer	GCTGAGTGCCAAGGATTT
NSUN4-Reverse Primer	GGAGGGAAGCGACTGATA
CD74-Forward Primer	TTATCTCCAACAATGAGCAACT
CD74-Reverse Primer	ACAGGAAGTAGGCGGTGGT
GAPDH-Forward Primer	AGAAGGCTGGGGCTCATTTG
GAPDH-Reverse Primer	AGGGGCCATCCACAGTCTTC

Table S4. Basic clinical information of the SLE patients (n=52).

No.	Age	Gender	Affected organs	Activity/ SLEDAI	Duration/yrs	Medications	$CD7^{high}CD74^{high}$Tex%	group
1	24	Female	skin	4	1	Hydroxychloroquine (HCQ)	19.60	Low
2	31	Male	skin, kidney, pericardium	11	2.5	Cyclophosphamide (CTX)	21.93	High
3	35	Female	skin, kidney	7	3	HCQ	19.64	Low
4	37	Female	skin, kidney, pericardium	13	2	HCQ+CTX+Co-Q10	10.23	Low
5	36	Male	skin, kidney, retina	14	2	HCQ+CTX	22.01	High
6	28	Female	skin, kidney	6	3	HCQ	23.69	High
7	40	Female	skin	2	0.5	HCQ	19.38	Low
8	27	Male	skin, kidney	8	1.5	HCQ	22.30	High
9	40	Male	skin, kidney	9	2.5	HCQ	31.45	High
10	28	Female	skin, kidney, joint	11	2	HCQ+CTX+Glucosamine Hydrochloride (GAH)	26.08	High
11	44	Female	skin	3	1	HCQ	12.21	Low
12	46	Female	skin, kidney, joint	12	2	HCQ+CTX+GAH	21.44	High
13	52	Female	skin, kidney	7	3	HCQ	25.03	High
14	29	Female	skin, kidney	6	1.5	HCQ	19.67	Low
15	48	Male	skin, kidney	11	2.5	HCQ	27.43	High
16	50	Male	skin, kidney, muscle, joint	14	3.5	HCQ+CTX+GAH	22.43	High
17	47	Female	skin, kidney	5	1	HCQ	16.51	Low

18	33	Female	skin, kidney, brain	13	4	HCQ+CTX+ Carbamazepine (CBZ)	25.66	High
19	34	Female	skin, kidney	6	2	HCQ	20.76	High
20	45	Female	skin, kidney	10	3	HCQ+CTX	21.15	High
21	43	Female	skin, kidney	10	1.5	HCQ+CTX	12.47	Low
22	31	Female	skin, kidney	13	5	HCQ+CTX	25.34	High
23	26	Male	skin, kidney	16	6	HCQ+CTX	26.05	High
24	49	Female	skin, kidney	4	0.5	HCQ	12.59	Low
25	44	Male	skin	5	2	HCQ	20.49	High
26	48	Female	skin, kidney, heart	18	8	HCQ+CTX+Co-Q10	27.56	High
27	38	Female	skin, kidney	6	2	HCQ	20.45	High
28	49	Female	skin, kidney, muscle	20	10	HCQ+CTX+Co-Q10	26.12	High
29	48	Male	skin, kidney	5	1.5	HCQ	8.08	Low
30	29	Female	skin, kidney, brain	23	8	HCQ+CTX+CBZ	26.54	High
31	50	Female	skin, kidney, brain	24	13	HCQ+CTX+CBZ	23.44	High
32	47	Male	skin	2	0.5	HCQ	9.34	Low
33	51	Female	skin, kidney, brain	19	7.5	HCQ+CTX+CBZ	27.34	High
34	52	Female	kidney	6	1.5	HCQ	12.54	Low
35	25	Male	skin	3	1	HCQ	11.69	Low
36	35	Female	skin, kidney, joint	16	5	HCQ+CTX+GAH	24.24	High
37	43	Female	kidney	4	1	HCQ	17.47	Low
38	34	Male	skin	2	2	HCQ	16.82	Low
39	37	Female	skin, kidney, retina	17	6	HCQ+CTX	21.87	High
40	36	Female	skin	1	2	HCQ	20.24	High

41	50	Male	skin	3	1.5	HCQ	13.43	Low
42	48	Female	skin, kidney	6	2	HCQ	20.96	Low
43	41	Female	skin, kidney	17	5.5	HCQ+CTX	26.84	High
44	44	Male	skin, kidney, joint	17	6.5	HCQ+CTX+GAH	22.99	High
45	45	Female	skin, kidney	6	2	HCQ	17.36	Low
46	54	Female	skin	1	2.5	HCQ	16.11	Low
47	26	Male	skin	3	1.5	HCQ	13.09	Low
48	42	Female	skin, kidney	12	3	HCQ+CTX	24.87	High
49	39	Female	skin, kidney, brain	22	11	HCQ+CTX+CBZ	23.86	High
50	41	Male	skin, kidney	5	2.5	HCQ	15.27	Low
51	32	Female	skin, kidney	6	1	HCQ	7.92	Low
52	49	Female	skin, kidney, heart, brain	23	10	HCQ+CTX+Co-Q10+CBZ	26.36	High

Table S5. The association of $CD7^{high}CD74^{high}$Tex% with the clinical indicators of the SLE patients.

**Clinical indicators**	No. of patients	High Tex (>20%)	adj-χ^2-value	p-value
**Age distribution**	52	30	3.512	0.061
≤ 40	25	14		
> 40	27	16		
**Sex**			15.022	**0.0001*****
Male	16	9		
Female	36	21		
**SLEDAI score**			7.031	**0.008****
< 10	28	8		
≥ 10	24	22		
**Duration**			0.300	0.584
< 5 years	39	17		
≥ 5 years	13	13		

Supplementary methods

Single-cell RNA-seq library preparation and sequencing

The cells were resuspended at a concentration of 700 cells/mL and immediately mixed with the same sample types immediately according to the 10X Genomics Chromium single-cell protocol for the v2 reagent kit (10X Genomics). Cell suspensions were loaded onto a chromium single-cell chip, along with a reverse transcription (RT) master mix and 30 gel beads. Single-cell gel beads were generated in an emulsion (GEMs). RT was performed using a C1000 Touch^{TM} Thermal Cycler (Bio-Rad) using the manufacturer's standard parameters. The cDNA was amplified and purified using SPRISelect beads (Beckman Coulter). Single-cell 30 libraries were then constructed following fragmentation, end repair, polyA tailing, adaptor ligation and size selection. Single-cell sequencing libraries were generated with one sample index for each sample and sequenced on an Illumina HiSeq X-Ten platform.

m5C-seq sequencing

Quality control and quantification of the constructed sequencing library were performed using the BioAnalyzer 2100 system (Agilent Technologies, USA), followed by 150 bp double ended sequencing on an Illumina NovaSeq 6000 to obtain the raw data. The Q30 value was then used for raw data quality control. The Cutadapt software (v1.9.3) was used to remove connectors, remove low-quality reads, and obtain high-quality clean reads. Hisat2 software was used to compare clean reads to the reference genome and HTSeq software (v0.9.1) was used to obtain the original count number. EdgeR was used for the standardization and calculation of multiple changes and p-values between the two groups of samples to screen differentially expressed genes. GO functional and KEGG pathway analyses were applied to the functional annotation of differentially expressed mRNA. The RNA m5C methylation sequencing service was provided by the Shanghai Yunxu Biotechnology Co., Ltd. (Shanghai, China). Immunoprecipitation was performed using GenSeq's m5C-IP kit, and the process was outlined as follows: RNA was randomly fragmented into approximately 200 nt fragments. Protein A/G magnetic beads were rotated and incubated with m5C antibodies at room temperature for 1 h to bind the antibodies to the magnetic beads. The RNA fragment was then incubated with the antibody bound to the magnetic beads for 4 h by rotating it at 4 °C, allowing the RNA to bind to the antibody. The combined complex was washed several times, and RNA was eluted from the complex. The Low Input Whole RNA Library Prep Kit (GenSeq, Inc.) was used to construct an RNA sequencing library. The constructed library was subjected to quality control using an Agilent 2100 biological analyzer, and high-throughput sequencing was performed on an Illumina NovaSeq sequencer. Q30 was used for quality control. Cutadapter software (v1.9.3) was used to remove low-quality reads and obtain high-quality clean reads, and Hisat2 software (v2.0.4) was used to match the clean reads of all samples to the human reference genome (HG38). MACS software was used to identify the methylated genes in each sample, and diffReps software was used to identify

differentially methylated genes. Finally, peaks located in the mRNA exon were screened and annotated. GO functional and KEGG pathway analyses were applied to the functional annotation of the differentially m5C modified mRNA.

Western blotting

Total protein was extracted from cells and tissues using radioimmunoprecipitation assay (RIPA) lysis buffer (Beyotime, Shanghai, China) and the concentration was measured using the BCA Protein Assay Kit (Beyotime). Protein samples were separated by SDS-PAGE at 70 V for 30 min, followed by 120 V for 90 min. The protein blots were transferred onto polyvinylidene fluoride (PVDF) membranes (Millipore, Bedford, MA, USA) at 300 mA for 2 h. After blocking with 5% nonfat milk for 2 h at room temperature, the membranes were incubated with the following primary antibodies (overnight at 4°C): NSUN4 (1:300, Abcam, ab235430), PD-1 (1:500, Abcam, ab309361), CTLA-4 (1:400, Abcam, ab231949), TIM3 (1:400, Abcam, ab47997), DRP1 (1:300, Abcam, ab184247), FIS1 (1:400, Abcam, ab156865), MFN1 (1:200, Abcam, ab221661), PINK1 (1:400, Abcam, ab216144), Parkin (1:400, Abcam, ab73015), COX IV (1:800, Abcam, ab14744) and β-tubulin (1:800, Abcam, ab176560). After incubation with horseradish peroxidase (HRP)-conjugated IgG (1:1500, Abcam, ab6721) at 37°C for 1 h. The protein bands were visualized using Enhanced chemiluminescence reagents (Millipore) in a Gel Imaging System (Thermo Fisher Scientific), and the relative levels of the proteins were analyzed using ImageJ software (Thermo Fisher Scientific).

Enzyme linked immunosorbent assay (ELISA)

Secretion of IFN-γ, IL-2 and TNF-α in the CD+ T cell supernatant was detected using the Human IFN gamma ELISA Kit (Abcam, ab174443), Human TNF alpha ELISA Kit (Abcam, ab181421) and Human IL-2 ELISA Kit (Abcam, ab270883), respectively, according to the manufacturer's protocols.
